# Longitudinal analysis of genetic and epigenetic changes in human pluripotent stem cells in the landscape of culture-induced abnormality

**DOI:** 10.1038/s12276-024-01334-8

**Published:** 2024-11-01

**Authors:** Yun-Jeong Kim, Byunghee Kang, Solbi Kweon, Sejin Oh, Dayeon Kim, Dayeon Gil, Hyeonji Lee, Jung-Hyun Kim, Ji Hyeon Ju, Tae-Young Roh, Chang Pyo Hong, Hyuk-Jin Cha

**Affiliations:** 1 College of Pharmacy, Seoul National University, Seoul, Republic of Korea; 2 College of Pharmacy and Research Institute of Pharmaceutical Sciences, Seoul National University, Seoul, Republic of Korea; 3 Department of Life Sciences, Pohang University of Science and Technology (POSTECH), Pohang, Republic of Korea; 4 Theragen Bio, Co., Ltd., Seongnam, Republic of Korea; 5 Korea National Stem Cell Bank, Cheongju, Republic of Korea; 6 Division of Intractable Disease Research, Department of Chronic Disease Convergence Research, Korea National Institute of Health, Osong Health Technology Administration Complex, Cheongju, Republic of Korea; 7 College of Pharmacy, Ajou University, Suwon, Republic of Korea; 8 YiPSCELL Inc., Seoul, Republic of Korea; 9 Catholic iPSC Research Center, College of Medicine, The Catholic University of Korea, Seoul, Republic of Korea; 10 Division of Rheumatology, Department of Internal Medicine, Institute of Medical Science, College of Medicine, The Catholic University of Korea, Seoul St. Mary’s Hospital, Seoul, Republic of Korea; 11 Department of Life Sciences, Ewha Womans University, Seoul, Republic of Korea; 12 Research Institute of Pharmaceutical Sciences, Seoul National University, Seoul, Republic of Korea

**Keywords:** Induced pluripotent stem cells, Gene expression

## Abstract

Human embryonic stem cells (hESCs) are naturally equipped to maintain genome integrity to minimize genetic mutations during early embryo development. However, genetic aberration risks and subsequent cellular changes in hESCs during in vitro culture pose a significant threat to stem cell therapy. While a few studies have reported specific somatic mutations and copy number variations (CNVs), the molecular mechanisms underlying the acquisition of ‘culture-adapted phenotypes’ by hESCs are largely unknown. Therefore, we conducted comprehensive genomic, single-cell transcriptomic, and single-cell ATAC-seq analyses of an isogenic hESC model displaying definitive ‘culture-adapted phenotypes’. We found that hESCs lacking TP53, in which loss-of-function mutations were identified in human pluripotent stem cells (hPSCs), presented a surge in somatic mutations. Notably, hPSCs with a copy number gain of 20q11.21 during early passage did not present ‘culture-adapted phenotypes’ or *BCL2L1* induction. Single-cell RNA-seq and ATAC-seq analyses revealed active transcriptional regulation at the 20q11.21 locus. Furthermore, the induction of *BCL2L1* and *TPX2* to trigger ‘culture-adapted phenotypes’ was associated with epigenetic changes facilitating TEA domain (TEAD) binding. These results suggest that 20q11.21 copy number gain and additional epigenetic changes are necessary for expressing ‘culture-adapted phenotypes’ by activating gene transcription at this specific locus.

## Stem Cell Therapy Risks: Unveiling Genetic Adaptations in hESCs

Human pluripotent stem cells (hPSCs) hold significant potential in regenerative medicine due to their ability to produce all types of cells. This study examines how genetic changes in hPSCs impact their stability and safety. Researchers used a set of identical human embryonic stem cells, grown for up to six years, and analyzed them using whole genome sequencing and single-cell sequencing techniques. The study found that certain genetic aberrations, especially mutations in the TP53 gene and a recurrent gain at 20q11.21, become more frequent as hPSCs are grown longer. These findings emphasize that TP53 mutations and 20q11.21 gains can alter the biological characteristics of hPSCs, affecting their safety more than the number of times the cells have been replicated. Understanding these changes is important for establishing guidelines to ensure the safety of stem cell therapy.

This summary was initially drafted using artificial intelligence, then revised and fact-checked by the author.

## Introduction

Owing to their unique pluripotency, human pluripotent stem cells (hPSCs) are a primary cell source for stem cell-based regenerative therapy. Owing to extensive preclinical research, many clinical studies on Parkinson’s disease, macular degeneration, heart failure, and some cancers are underway^1^. However, other than potential teratoma formation by unintended residual undifferentiated hPSCs, which has been extensively reviewed^2^, unexpected genetic aberrations in hPSCs^3^ are the predominant technical hurdles for ensuring safe stem cell therapy due to their clinical uncertainty^4^.

Considerable attention has been given to elucidating the biological consequences, risk thresholds, and causes of hPSC genetic instability, as numerous studies have reported recurrent copy number variations (CNVs) in various chromosomes, including 20q11.21^4^. Genetic hPSC aberrations vary from CNVs, somatic mutations, and chromosome alterations (e.g., trisomy at chromosomes 12 and 17)^5,6^. Nevertheless, somatic hPSC mutations during in vitro culture are relatively rare^7^. Thus, the collapse of unique hPSC characteristics, especially their high susceptibility to genotoxic stimuli^8,9^ to preserve genome integrity, is closely associated with genetic aberrations. This observation may explain the frequent gains at the 20q11.21 locus containing *BCL2L1*^10^ to induce antiapoptotic BCL-xL protein^11–13^ and loss of 17p13.1, where TP53 is present^14^, and somatic TP53 mutations^15,16^ in hPSCs during prolonged in vitro culture. Interestingly, these genetic aberrations, such as the induction of BCL-xL as an ‘acquired survival trait’, serve to rescue cells from apoptosis triggered by aberrant mitosis, which often results from TPX2 induction in hPSCs^17,18^. This, in turn, leads to aneuploidy or additional aberrations^19^. Consequently, genetic alterations that enable survival under the selective pressures of in vitro culture conditions can potentially trigger further structural changes in the genome^20^. This hypothesis has been substantiated through retrospective studies with multiple hPSC lines collected from diverse research institutions^10,15,16^.

However, 20q11.21 gain alone is insufficient to drive precise phenotypic changes such as an ‘acquired survival advantage’^21^. Thus, additional cues would be necessary to induce *BCL2L1* and *TPX2* gene transcription, which yields clear ‘acquired survival traits’ through BCL-xL protein and YAP/TEAD4-dependent gene expression, respectively^11–13,17^. Recurrent epigenetic changes, such as hyper-/hypo-methylation, parental imprinting loss, and variable X chromosome inactivation, transpire during prolonged hPSC culture^22^. In particular, hypermethylation leads to the epigenetic repression of multiple antioxidant genes^23^ in prolonged hPSC cultures with abnormal karyotypes, ‘differentiation-related genes,’ and ‘tumor-suppressor genes’^24^. Therefore, isogenic hESC sets with varying durations during in vitro maintenance exhibit unique cellular and molecular characteristics, enhancing the monitoring of progressive deterioration.

This study used an isogenic human embryonic stem cell (hESC) set with different culture periods of up to six years to monitor stepwise epigenetic and genetic alterations during consecutive in vitro hESC cultures. Multiomics analysis, including whole-genome sequencing (WGS), single-cell RNA sequencing (scRNA-seq), and single-cell ATAC sequencing (scATAC-seq), revealed that dominant-negative TP53 mutations accentuated somatic mutations. In addition, epigenetic alterations at the 20q11.21 locus promoted gene expression at 20q11.21 through transcriptionally enhanced associate domain (TEAD)-dependent transcription. These results account for 20q11.21 gain and TP53 mutations, the most prevalent genetic aberrations in hPSCs.

## Materials and methods

Details of the methods are available in the online supplement.

### Cell line and culture

Human embryonic stem cells (WA09: H9, WiCell Research Institute) were maintained in iPSC-brew StemMACS (Miltenyi biotechnology, #130-104-368) with 0.1% gentamycin (Gibco, Waltham, MA, USA, #15750-060) on a Matrigel (Corning, Corning, NY, USA, #354277)-coated cell culture dish at 37 °C and humidified to 5% in a CO2 incubator. The media of the cells were changed daily, and the cells were passaged every 5–6 days. The cell density was kept under 80% and over 70% when subcultured, and the average cell count of hESCs at the time of maintenance was 6 ~ 8 × 10^5^ in each 60 mm dish. Accordingly, the initial seeding number did not exceed 10^5^. The doubling time of hESCs is 22 ~ 26 h under ordinary culture conditions. Upon transfer, the hESCs were rinsed with DMEM/F-12 (Gibco) and detached enzymatically with dispase (Life Technologies), followed by 3 washes with DMEM/F-12 (Gibco #11320-033) before plating. If needed, 10 µM Y27632 (Peprotech#1293823) was used for cellular attachment.

### Gene knockout and electroporation

gRNA for TP53 knockout (S: CCCTTCCCAGAAAACCTACCAGG, AS: CCTGGTAGGTTTTCTGGGAAGGG) was transfected via electroporation (NEPA-21) with a poring pulse of 175 V and a pulse length of 2.5 ms. The cells were detached with Accutase (BD Bioscience, 561527), washed and diluted with Opti-MEM (Gibco, 31985070) to a final concentration of 1 × 10^6^ cells/100 $\mu$L. After transfection, the cells were cultured for 1 week before single-cell picking and further validated by sequencing. The functional loss of p53 in p53KO single clones was validated by survival after Nutlin-3a treatment.

### Whole-genome sequencing and detection of somatic mutations

Genomic DNA was isolated from P1, P2, P3, and P4 hESCs via the Wizard® HMW DNA Extraction Kit (#A2920, Promega, USA) for whole-genome sequencing on the Illumina NovaSeq platform. Libraries were prepared from 1 µg of input DNA via a TruSeq DNA Sample Prep Kit according to the manufacturer’s instructions (Illumina, Inc., San Diego, CA, USA). The sheared DNA fragments underwent end repair, A-tailing, adapter ligation, and amplification, followed by cleanup. These libraries were then subjected to paired-end sequencing with a read length of 150 bp on the Illumina NovaSeq 6000 platform, yielding an average of 106.1 Gb per library.

Clean reads from each sample, exhibiting a quality of >Q30 (%), were aligned to the human reference genome (GRCh37/hg19) via BWA (version 0.7.17). PCR duplicates were identified and removed via MarkDuplicates of the Picard tool (version 2.25.5). To optimize read mapping quality, base quality score recalibration was conducted via the BaseRecalibrator tool in GATK (version 4.1.9.0). Somatic mutations in P2, P3, and P4 hESCs were detected via MuTect2 (version 4.1.8.1), with P1 hESCs serving as the reference. High-confidence somatic mutations were characterized as the subset of somatic variant calls fulfilling the following criteria: (1) sites with a VAF of 0 in P1 hESCs, (2) biallelic SNVs, (3) mapped read depth of ≥20, (4) a minimum alternate read depth of ≥3, (5) exclusion of germline-like heterozygous SNVs, and (6) exclusion of SNVs on the X and Y chromosomes. The identified somatic variants were annotated with SnpEff (version 4.3) and cross-referenced against the Catalog of Somatic Mutations In Cancer (COSMIC) and The Cancer Genome Atlas (TCGA) databases.

### Evaluating tumorigenic potential in hESCs with missense mutations in TP53-linked genes

To assess the extent to which missense mutations occur in TP53-linked genes, we developed a scoring system based on two hypotheses. First, we assumed that the impact of a mutation is related to the frequency of inter-amino acid changes. For example, the KRAS-G12D mutation, which is known to cause tumors, involves a change from neutral glycine to negative aspartic acid. According to the BLOSUM100 matrix, this mutation occurs naturally with approximately 1.8% probability. Therefore, we derived scores for amino acid changes on the basis of the BLOSUM100 matrix for mutations occurring in sequences with 100% similarity. We summed the scores for amino acid changes with negative values, assuming that changes occurring frequently would not affect the gene’s function, to create the BLOSUM score (B score) (Eq. 1).

1 $${B}_{A}=-\mathop{\sum }\limits_{i=1}^{n}I({BLOSUM}100\left({Ref},{Alt}\right))[I\left(x\right)=0\,{if}\,x\ge 0],$$

where ${B}_{A}$ is the B score of sample A. *BLOSUM100* (*Ref*, *Alt*) represents the BLOSUM100 score for amino acid transition between the reference amino acid (Ref) and altered amino acid (Alt). *I(x)* is an indicator function that returns 0 when there are positive values in the inter-amino acid change according to the BLOSUM100 matrix.

On the other hand, there are mutations such as TP53-R135H, a well-known tumorigenic TP53 hotspot mutation, that do not follow the first hypothesis, where both R and H are neutrally charged and occur with approximately 27% probability. To complement this, the second hypothesis introduces the possibility that the mutation is a hotspot mutation. For this scoring system, we obtained *p* values from a study by Gao et al.^25^. We transformed these *p* values into a log_2_ scale, and if the mutation matched Gao’s defined list, we summed the log_2_ (*p* value) values to create the Hotspot score (H score) (Eq. 2).

2 $${H}_{A}=-\mathop{\sum }\limits_{k=1}^{n}{\log }_{2}{P}_{k}[k\in {Ga}{o}^{{\prime} }s{hotspot\; mutation}],$$

where ${H}_{A}$ is the H score of sample A and where ${P}_{k}$ represents the *p* values of genes that have Gao hotspot mutations.

Finally, the ${{TP}53{score}}_{A}$ for sample A’s TP53-linked gene was calculated by summing the B score and H score and then passing the value through the hyperbolic tangent function to produce a standardized score ranging from 0 to 1 (Eq. 3).

3 $${{TP}53{score}}_{A}=\tanh ({B}_{A}+{H}_{A}),$$

### Copy number variation detection in whole-genome sequence data

CNVs were identified in the whole-genome sequence data for P2, P3, and P4 hESCs via CNVKit (version 0.9.10), with P1 hESCs serving as the reference. CNVs exhibiting significant gains or losses were selected, applying a stringent cutoff of an absolute copy number ratio exceeding 0.85. Furthermore, CNVs identified in the whole-genome sequence data were confirmed via the CytoScan HD array (Thermo Fisher Scientific) or array comparative genomic hybridization (array CGH) (Thermo Fisher Scientific).

### Bulk RNA-seq and bioinformatic analysis

cDNA libraries were prepared from 1 μg of total RNA from each sample via the TruSeq Stranded mRNA Sample Prep Kit (Illumina, Inc., San Diego, CA, USA) according to the manufacturer’s instructions. After qPCR validation, the libraries were subjected to paired-end sequencing with a 150 bp read length via an Illumina NovaSeq 6000 platform, yielding an average of 5.9 Gb per library. Clean reads with quality scores of >Q30 were aligned to the human reference genome (GRCh37/hg19) via STAR (version 2.7.1a) with the default parameters. Gene expression quantitation was performed via RSEM (version 1.3.1). The number of transcripts per kilobase million (TPM) was calculated as the expression value. The differentially expressed genes (DEGs) were identified via DESeq2 (version 1.26.0), with the cutoff set at *q* < 0.05 and >1.5-fold change. Gene Ontology (GO) enrichment analysis for DEGs was conducted via the DAVID Gene Functional Classification Tool (http://david.abcc.ncifcrf.gov; version 6.8), with an EASE score cutoff of <0.01.

### Single-cell RNA sequencing (scRNA-Seq) and bioinformatic analysis

Single-cell suspensions with 90% cell viability were processed on a 10x Chromium Controller via Chromium Next GEM Single Cell 3′ Reagent Kits v3.1 (10x Genomics) according to the manufacturer’s instructions. The cells were partitioned into nanoliter-scale gel beads-in-emulsion (GEMs) with a target recovery of 10,000 cells. The single-cell 3 prime mRNA-seq library was generated via reverse transcription, cDNA amplification, fragmentation, and ligation with adapters followed by sample index PCR. The resulting libraries were quality checked via a Bioanalyzer and sequenced on an Illumina NovaSeq 6000 (index = 8 bases, read 1 = 26 bases, and read 2 = 91 bases). We generally acquired an average of 516.1 million reads per library. Paired-end sequencing reads were processed via Cell Ranger (10x Genomics software, version 1.3.1). The reads were aligned to the human reference genome (GRCh37/hg19) for demultiplexing, barcode filtering, gene quantification, and gene annotation. Barcodes with less than 10% of the 99th percentile of total unique molecular identifier (UMI) counts per barcode, which are likely to be empty droplets, were removed. Following this quality control step, gene barcode matrices for each sample were generated by counting the number of UMIs for a given gene in the individual cell. To analyze the heterogeneous composition of the cell populations, gene barcode matrices were processed with the Seurat R package (version 2.4). Low-quality cells with fewer than 1000 or more than 7200 detected genes were removed. In addition, cells whose mitochondrial gene content was >11% were also removed. After low-quality cells were filtered out, the gene expression values were naturally log-transformed and normalized to the sequencing depth scaled by a multiplier (e.g., 10,000). To reduce the variance caused by unwanted sources, variations in gene expression driven by cell cycle stages (S and G2/M phases) and mitochondrial gene expression were regressed out via the vars.to.regress argument in the ScaleData function of Seurat. Next, unsupervised clustering analysis of the scRNA-seq dataset was performed. Highly variable genes were selected via the FindVariableFeatures function in Seurat, and dimensionality reduction of the data was performed by computing the significant principal components of highly variable genes. Unsupervised clustering was performed by using the FindClusters function in Seurat with the resolution argument set to 0.3, and the resulting clusters were then visualized in a uniform manifold approximation and projection (UMAP) plot. After unsupervised clustering, DEGs among each cell cluster were identified via the FindAllMarkers function in Seurat. Additionally, significant DEGs were filtered based on the following criteria: (1) *p* < 0.001, (2) a fold change greater than an absolute value of 0.584 on a log2 scale, and (3) differences in expression between pct1.1 and pct.2 exceeding 0.3. To annotate the biological process functions of DEGs from clusters, GO enrichment analysis was conducted via the DAVID Gene Functional Classification Tool (http://david.abcc.ncifcrf.gov; version 6.8), applying a cutoff of an EASE score <0.01. We also used the Monocle3 R package to reconstruct cellular trajectories by computing and ordering the sequence of gene expression changes in cells collected from different time points (hESCs passaged in vitro < 50 or approximately 100, 200, and 300 times). First, DEGs were identified via the differentialGeneTest function with *q* < 0.01 in Monocle3. The dimensions of the data were reduced via discriminative dimensionality reduction with trees (DDRTree). Next, the cells based on the selected DEGs were ordered via the orderCells function in Monocle3, and the trajectory of the cells was visualized via the plot_cell_trajectory function in Monocle3.

### Single-cell ATAC-seq library preparation and bioinformatic analysis

Nuclei were isolated from cells via the Nuclei Isolation Kit: Nuclei EZ Prep (NUC101-1KT) according to the manufacturer’s instructions. The sequencing library was prepared via the Chromium Next GEM Single-Cell ATAC Reagent Kit (10x Genomics; PN-1000176, v1.1). Approximately 8000 nuclei were isolated for Tn5 transposition, nuclear barcoding, and library construction. The quality-checked libraries were sequenced via the Illumina sequencing platform. The paired sequencing reads and barcodes were demultiplexed, preprocessed, aligned to the hg38 human reference genome and used to call peaks via the single-cell ATAC Cell Ranger pipeline (version 2.0.0). The resulting matrices were further analyzed via the R package Signac (version 1.4.0)^26^. Clustering cells and finding differentially accessible regions (DARs) were performed with integrated samples. DARs were selected with the following thresholds: false discovery ratio <0.01 and log_2_-fold change >0.3. All collected DARs among major clusters were grouped via K-means clustering with k = 10. A cis-coaccessibility network (CCAN) was built via Cicero (version 1.3.4.11), with a coaccessibility score >0.05 as the threshold^27^. Genes with an upstream 1 kb region included in the CCAN were identified as candidate targets for DARs. A functional enrichment assay of candidate target genes was performed via Metascape^28^. The TF activity score was calculated via the RunChromVAR() function^29^ with the JASPAR2020 database^30^. The AUC score was calculated via the FindMarkers() function. To profile each cell cluster, subset bam (version 1.1.0) was used to separate the read pairs from the integrated sequencing data. Peak calling for each cluster was performed via MACS2 (version 2.2.9.1)^31^. ATAC-seq footprints were analyzed within peaks via HINT-ATAC (version 1.0.2)^32^. DeepTools (version 3.1.3)^33^ was used to calculate the fold change in the ATAC-seq signal compared with the P1 sample and to draw heatmaps around the peak centers. To compare ATAC and CNV, the patterns of changes in ATAC and CNV were calculated across each gene body via log_2_-fold change against P1. The similarity scores of ATAC and RNA were calculated via the inverse of the Euclidean distance between the patterns of ATAC and RNA.

## Results

### The number of drastic somatic mutations increases in culture-adapted hESCs

**Fig. 1:**
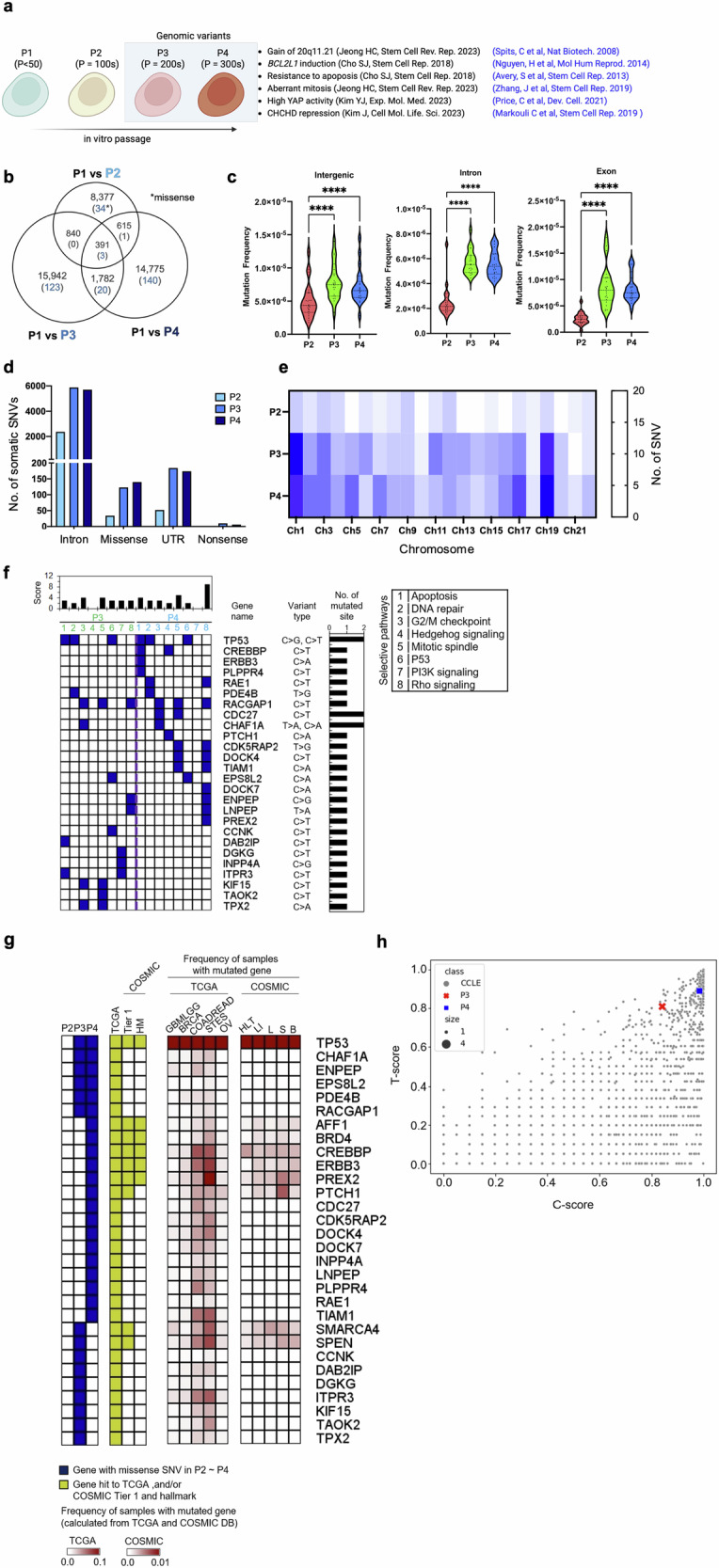
The number of drastic somatic mutations increases in culture-adapted hESCs. **a** General scheme of passage-dependent isogenic pairs of hESCs. P1 hESCs: fewer than 50 passages (one-year culture), P2 hESCs: more than 100 passages (two-year culture), P3 hESCs: more than 200 passages (four-year culture), and P4 hESCs: more than 300 passages (culture greater than six years). **b** Number of somatic single-nucleotide variants (SNVs) detected by whole-genome sequencing of P1, P2, P3 and P4 hESCs and overlaps of somatic SNVs (detected via Mutect2) among those hESCs. The number of missense SNVs is highlighted in blue. **c** The frequencies of somatic SNVs in intergenic regions, introns, and exons in P2, P3 and P4 hESCs were calculated by dividing the number of somatic SNVs found in each genomic feature by the length of that region. **d** The number of somatic intron, missense, 5′/3′-UTR, and nonsense SNVs identified in P2, P3, and P4 hESCs. **e** The chromosomal distribution of somatic SNVs in P2, P3, and P4 hESCs. The abundance of somatic SNVs is presented as a heatmap. **f** Functional pathways involved in the functions of survival and proliferation in stem cells and mutated in P3 and P4 were analyzed via Qiagen Ingenuity Pathway Analysis, as described in the supplementary methods. **g** Genes with somatic missense SNVs (highlighted in blue in the first panel of Fig. 1g) were checked against The Cancer Genome Atlas (TCGA) and the Catalog of Somatic Mutations in Cancer (COSMIC)-Tier 1 and -hallmark (HM) databases (highlighted in yellow in the second panel of Fig. 1g). **h** Somatic missense SNVs in P3 and P4 hESCs were searched against the TP53-directed network and/or COSMIC Tier 1 datasets, and the impact of those mutations was assessed via T scores for the TP53-direct network and C-scores for COSMIC Tier 1 (see the Methods). Finally, the resulting scores were compared with data from the Cancer Cell Line Encyclopedia (CCLE).

We identified the cellular and molecular events that occur during long-term in vitro passage using H9 hESCs with different passage numbers, which were maintained for up to 6 years (Fig. 1a). Genomic variants (i.e., P3 and P4 hESCs) share typical ‘culture-adapted phenotypes’, as previously demonstrated^11,12,19,34–36^ and depicted in Fig. 1a. As these ‘culture-adapted phenotypes’ were manifested in P3 hESCs (but not P2 hESCs) and P4 hESCs with an additional 17q24 gain^18^ (Supplementary Fig. 1a) over P3 hESCs (with a 20q11.21 gain), closer examination of ‘stepwise variation models for genome hESC instability’ is warranted^20,37^.

We investigated progressive somatic mutations in long-term culture-adapted hESCs by performing WGS on the hESC set and analyzing the genomic distribution of somatic single-nucleotide variants (SNVs) in P2, P3, and P4 hESCs, with P1 as a normal control. Following quality control guidelines (see the Materials and Methods section), 42,722 somatic SNVs were identified. The primary variant types analyzed were C > T, C > A, and T > C (Supplementary Fig. 1b). Somatic SNVs in P3 and P4 hESCs increased by approximately 1.8-fold compared with those in P2 hESCs, which coincides with the significant occurrence of P3- and P4-specific SNVs. (Fig. 1b). This correlation demonstrated an association between the increase in passage number and somatic mutation.

Intergenic, intronic, and exonic region mutation frequencies, as annotated in the GRCh37 genome assembly, were measured and normalized to the base-pair content of each feature class (Fig. 1c, Supplementary Fig. 1b, c). P2 and P3/P4 hESC mutation frequencies significantly differed in introns (approximately 2.53 [*P* = 2.25 × 10^−8^] and 2.47 [*P* = 3.26 × 10^−9^] times more abundant in P3 and P4 than in P2, respectively) and exons (approximately 4.10 [*P* = 4.58 × 10^−8^] and 4.36 [*P* = 1.07 × 10^−8^] times more abundant in P3 and P4 than in P2, respectively). Specifically, concerning coding regions, a significant increase in missense and nonsense variants was observed in P3 and P4 (Fig. 1d). Our results indicate that genetic variant accumulation in genic regions during long-term in vitro passaging augments hESC genome instability.

Interestingly, we observed that the somatic mutations were nonrandom; somatic SNVs in chromosomes 1 and 19 were the most prevalent (Fig. 1e and Supplementary Fig. 1d). Notably, recurrent gains are frequently observed on chromosome 1^38,39^. Along with the drastic increase in P3 and P4 hESC mutations, we identified 29 missense mutations, primarily consisting of C > T and C > A variant types (Supplementary Fig. 1c), in 26 genes associated with apoptosis, DNA repair, the G2/M checkpoint (cell cycle), the mitotic spindle, P53, Hedgehog signaling, PI3K signaling, and Rho signaling (Fig. 1f). Among these pathways, PI3K gain and Rho signaling loss also increase the survival benefit of hPSCs according to unbiased genome-wide screening^40^ or genetic perturbation studies^41^.

We identified 30 genes with missense SNVs in P3 or P4 hESCs associated with tumorigenesis in The Cancer Genome Atlas (TCGA) (second panel in Fig. 1g). These genes were frequently localized in colon and rectum adenocarcinomas in the TCGA cohort (third panel in Fig. 1g). Additionally, nine of these genes, including *TP53*, *AFF1*, *BRD4*, *CREBBP*, *ERBB3*, *PREX2*, and *PTCH1*, were also prevalent in the Catalog of Somatic Mutations in Cancer (COSMIC)^42^ Tier 1. COSMIC collects genes contributing to tumor promotion and directly accounts for clinical PSC cell therapy risks (second and fourth panels in Fig. 1g). To further assess tumorigenesis potential in P3 and P4 hESCs, we scored the impact of missense SNVs in genes within the TP53 network (T score) or COSMIC Tier 1 (C score) based on the Cancer Cell Line Encyclopedia (CCLE; Fig. 1h). The assessment revealed high tumorigenesis potential in P3 and P4 hESCs, as indicated by their high T- and C-scores, where the closer the score was to 1, the greater the impact was (Fig. 1h).

### TP53 mutations relative to an increase in somatic mutations

**Fig. 2:**
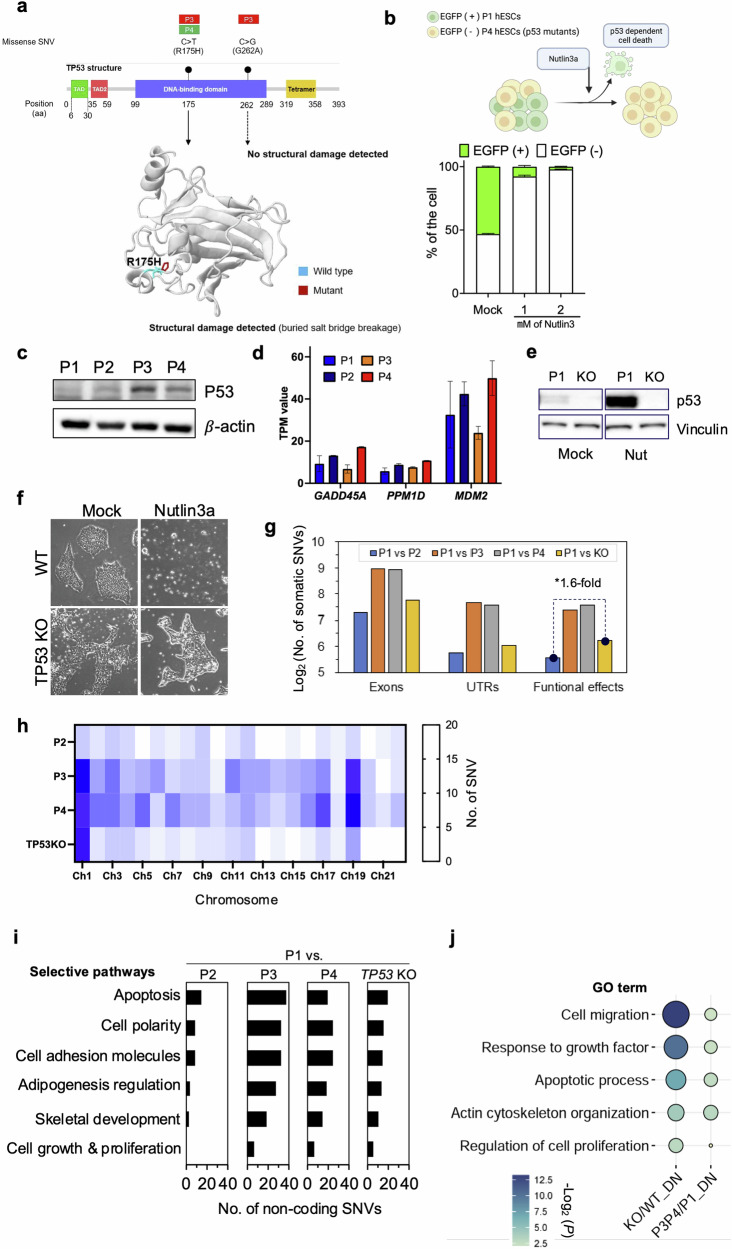
TP53 mutations relative to an increase in somatic mutations. **a** The identification of somatic missense SNVs in TP53 of P3 and P4 hESCs and the structural changes introduced by the missense SNV (R175H). Structural changes in TP53 were predicted via Missense3D. **b** Normal H9 cells tagged with eGFP and p53 mutant H9 were mixed and cultured. Nutlin-3a, a p53 activator, was added for 24 h, after which the cell populations were assayed and analyzed via flow cytometry. **c** Basal p53 protein levels in P1, P2, P3 and P4. **d** Expression levels of the p53 downstream genes *GADD45A*, *PPM1D* and *MDM2*. RNA-Seq for P1, P2, P3, and P4 hESCs was performed, and gene expression levels were quantified via RSEM with transcripts per million mapped reads (TPM) values. **e** p53 protein levels in established TP53KO cells compared with those in p53 normal H9 cells were determined by immunoblotting after 16 h of 1 µM Nutlin-3a treatment. **f** Resistance of TP53KO H9 cells to the p53 activator Nutlin-3a compared with that of the isogenic Mock pair. Nutlin-3a (2 µM) was added for 24 h. **g** The number of somatic SNVs identified in exons of P1-derived *TP53* KO hESCs. In Fig. 2G, untranslated regions (UTRs) encompass both the 5′ and 3′ ends, and the functional effects include missense, nonsense, and splice sites. **h** The chromosomal distribution of somatic SNVs in P2-, P3-, P4-, and P1-derived *TP53*-KO hESCs. The abundance of somatic SNVs is presented as a heatmap. **i** Functional pathway annotation for genes with somatic noncoding SNVs in P2, P3, P4, and P1-derived *TP53* KO hESCs. The functional pathways involved in the survival and proliferation of stem cells related to culture adaptation were selectively analyzed via Qiagen Ingenuity Pathway Analysis. **j** Gene Ontology (GO) enrichment analysis of downregulated genes in *TP53*-KO hESCs compared with wild-type (WT or P1) hESCs. The GO enrichment results were performed and validated as described in the methods section.

Recent studies have highlighted the incidence of dominant-negative mutations in TP53 within hPSCs^15^. Since p53 is pivotal in inducing cell death in hPSCs^8,43^, p53 stabilization markedly promotes this outcome. Thus, with passaging, clonal dominance is readily achieved in TP53 mutant clones^14^. Two somatic missense SNVs, c.524 G > A (p.Arg175His for P3 and P4 hESCs) and c.785 G > C (p.Gly262Ala for P3 hESCs), were found in the *TP53* DNA-binding domain (DBD), where most *TP53* mutations develop in hPSCs^14^ (Fig. 2a). Remarkably, a missense SNV, c.524 G > A, predicted p53 structural damage (Fig. 2a). As previously described^15^, when mixed with P1 hESCs, P4 hESCs with TP53 mutations became dominant clones soon after treatment with Nutlin-3a (for inducing p53-dependent cell death) (Fig. 2b). This result corroborates the observation that p53 protein levels were markedly stabilized in P3 and P4 hESCs even without stimuli (Fig. 2c), with no significant TP53-dependent gene upregulation (i.e., *GADD45A, PPM1D*, or *MDM2*) (Fig. 2d). These findings demonstrate that TP53 mutations in P3 and P4 hESCs were dominant-negative.

In the following STRING network analysis^44^, numerous somatic mutations were identified in genes linked to mutated *TP53* in P3 and P4 hESCs (Supplementary Fig. 2a). This discovery suggests that mutated *TP53* may influence mutations in other gene nodes within the *TP53* network and increase the number of somatic mutations in P3 and P4 hESCs (Fig. 1d). Considering that p53 is integral for genome guidance^45^, we theorized that dominant-negative TP53 mutations favor the accumulation of somatic mutations, as depicted in Supplementary Fig. 2a. Therefore, we produced TP53 knockout hESCs (KO hESCs) from P1 hESCs (normal hESCs) by introducing an indel (insertion and deletion) in exon 4 with Cas9 (Supplementary Fig. 2b). Clonal selection was performed after Cas9 was used to establish TP53-KO hESCs (KO hESCs) with one base pair (bp) insertion to induce frameshift mutation (Supplementary Fig. 2c). Functional p53 KO was verified by the lack of p53 protein (Fig. 2e) and clear survival after Nutlin-3a treatment in KO hESCs (Fig. 2f).

Through WGS, we identified more exonic somatic mutations in KO hESCs than in P2 hESCs (Fig. 2g). Functional effect variants, including missense, nonsense, and splice-site SNVs (Supplementary Fig. 2d), were 1.6 times more abundant. The analysis also revealed a predominant distribution of somatic mutations on KO hESC chromosome 1, similar to P3 and P4 hESCs (Fig. 2h). This correlation suggests that TP53 mutations result in the accumulation of somatic mutations on specific chromosomes. Although most somatic mutations were identified in noncoding KO sequences, we conducted a functional annotation related to cell differentiation for genes with these mutations. The analysis revealed frequent gene mutations associated with functions such as apoptosis, cell polarity, cell adhesion, adipogenesis regulation, stemness, cytoskeletal development, and chromatin modification, which may be involved in the acquisition of hESC survival traits (Fig. 2i). In particular, genes related to apoptosis and growth were downregulated in TP53 KO cells (Fig. 2j). We also identified numerous somatic mutations in gene nodes within the TP53 KO network (Supplementary Fig. 2e), indicating somatic mutation expansion and accumulation within the TP53 network. A gain event with abnormal copy number changes throughout chromosome 1, with a significant copy number ratio, was also identified (Supplementary Fig. 2f, g). In contrast to P3 and P4 hESCs, TP53 KO hESCs did not exhibit CNV at 17q24.1/2 or 20q11.21 (Supplementary Fig. 2h). This result demonstrates that TP53 KO has undergone significant genetic alterations, affecting genetic instability.

### Culture-adapted hESC cellular heterogeneity and transcriptome profiles

**Fig. 3:**
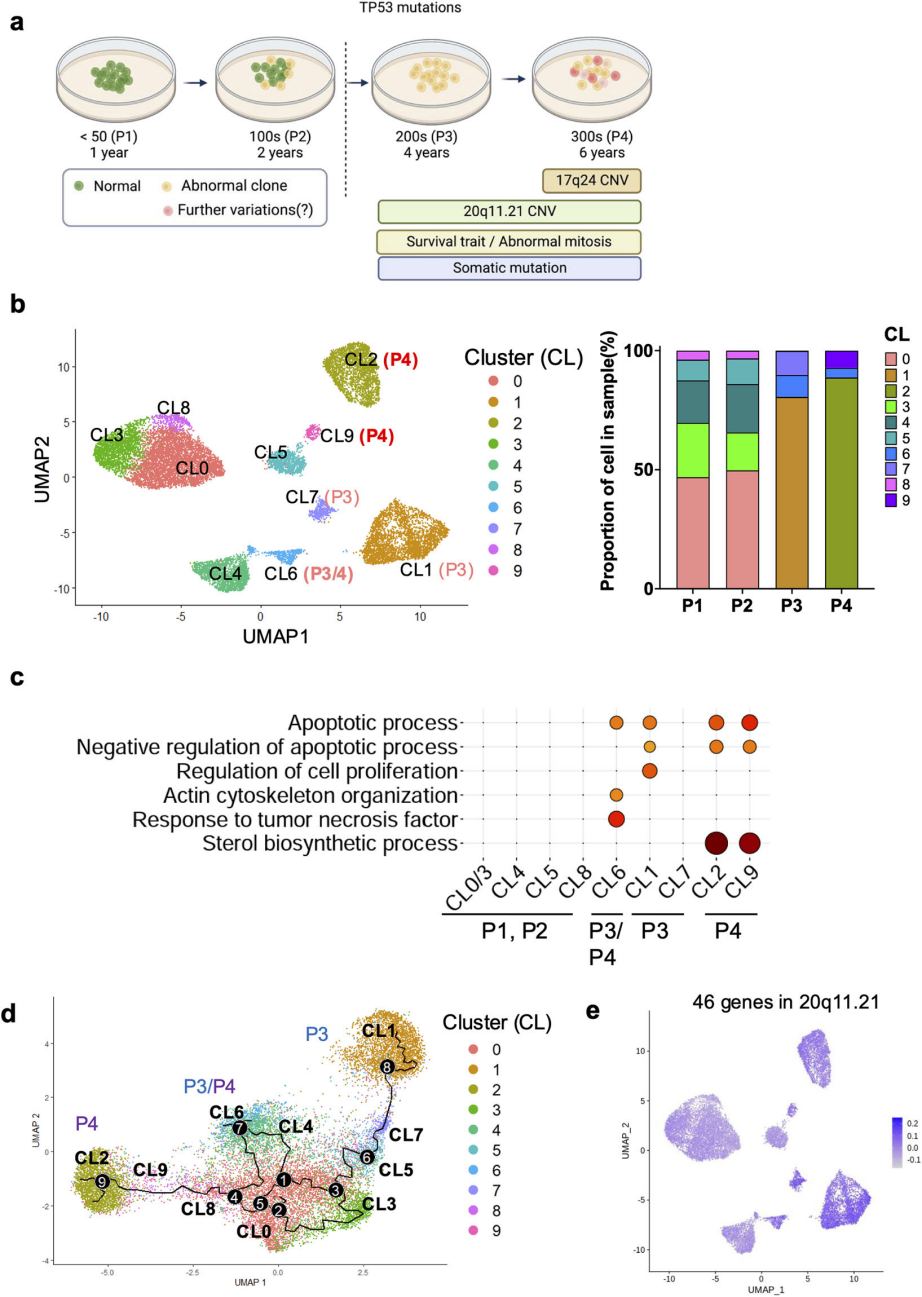

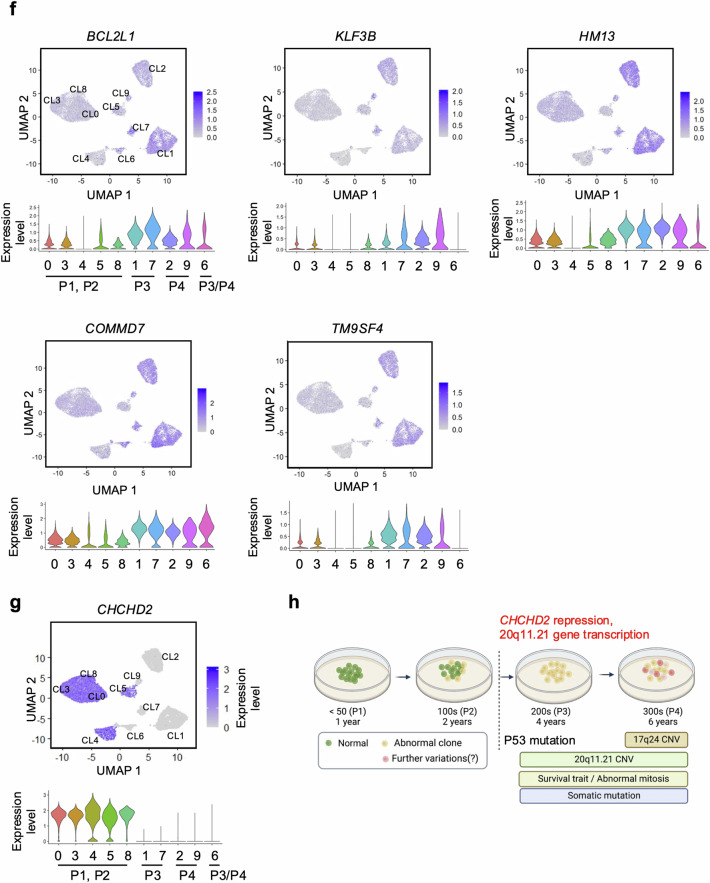
Culture-adapted hESC cellular heterogeneity and transcriptome profiles. **a** Scheme of isogenic pair hESCs and single-cell analysis considering the TP53 mutation status. **b** UMAP plot visualization of cultured hESCs (P1, P2, P3, and P4) colored according to 10 different transcriptionally distinct clusters (left panel); CL0, CL3, CL4, CL5 and CL8 for both P1 and P2 hESCs; CL1 and CL7 for P3 hESCs; CL2 and CL9 for P4 hESCs; and CL6 for both P3 and P4 hESCs. The right panel in Fig. 3b represents the composition of the clusters in each sample and the proportion of cells within each cluster. **c** GO terms enriched for each cluster based on differentially expressed genes. GO enrichment analysis was conducted via DAVID with a cutoff EASE score < 0.01. **d** Single-cell trajectory reconstructed by Monocle 3 for the four cultured hESCs. The trajectory indicates that P3 and P4 hESCs generated significant cellular heterogeneity from P1/P2 hESCs. Even between P3 and P4 hESCs, a high level of cellular divergence is shown. **e** Expression levels of 46 genes at 20q11.21 are indicated in each cell projected on the UMAP plot via the feature plot function. **f** Expression levels of *BCL2L1*, *KLF3B*, *HM13*, *COMMD7*, and *TM9SF4* at 20q11.21 are indicated in each cell projected on the UMAP plot via the feature plot function. The VlnPlot below the UMAP plot shows the distribution of single-cell gene expression in each cluster. The y-axis of each panel represents the expression levels of the indicated genes. **g** Expression of *CHCHD2* in Clusters 0, 3, 4, 5, and 8 for P1 and P2 hESCs. **h** Graphical representation of distinct changes in P3 and P4 hESCs, as determined by scRNA-seq (shown in red).

Despite the correlation between TP53 mutations and increased somatic mutations, TP53 mutations did not account for the 20q11.21 CNV gain and highly representative gene expression (*TPX2* and *BCL2L1*) to trigger typical cellular events for “culture adaptation” (i.e., abnormal mitosis and survival traits; data not shown) and further genetic aberrations (i.e., additional CNVs such as 17q24 gain), as shown in Supplementary Fig. 2h (Fig. 3a). To monitor the variations in long-term hESC cultures, transcriptome profiles from P1, P2, P3, and P4 hESCs were obtained at the single-cell level (Fig. 3b). The cellular divergence of P3 (Clusters 1, 6, and 7) and P4 (Clusters 2, 6, and 9) hESCs from P1 and P2 hESCs (Clusters 0, 3, 4, 5, and 8) was identified via UMAP clustering analysis (Fig. 3b). The gene ontology (GO) annotation for genes highly expressed in the P3 and P4 hESC clusters revealed a unique function associated with long-term culture adaptation. Gene signatures of the apoptotic process related to negative regulation were consistently altered in the P3 and P4 hESC clusters. Similarly, ‘sterol biosynthesis’ was significantly enriched in P4 hESC clusters (Fig. 3c). The unique enriched gene set in P4 hESCs compared with that in P3 hESCs implied that further variation from P3 hESCs would lead to the acquisition of unique P4 hESC characteristics. The RNA velocity analysis to determine each cluster’s trajectory supported this hypothesis (Fig. 3d). Intriguingly, the expression of genes at the 20q11.21 locus, which consists of 46 genes, was relatively increased in all P3 and P4 hESC clusters (Fig. 3e). The expression levels of *BCL2L1, KIF3B, HM13, TM9SF4* and *COMMD7*, which are involved in cell proliferation, differentiation inhibition, and anti-apoptosis^46^, at 20q11.21 were markedly elevated in P3 and P4 hESCs (Fig. 3f), indicating a potent selective advantage in culture.

One of the most distinct differences in gene expression between clusters of normal hESCs (Clusters 0, 3, 4, 5, and 8) and the variants (Clusters 1, 6, and 7 for P3; Clusters 2, 6, and 9 for P4) was the expression of *CHCHD2* (Fig. 3g). Notably, *CHCHD2* is sharply repressed in hESCs with a gain of 20q11.21^47^ or after frequent exposure to a ROCK inhibitor^41^. The active transcription at the 20q11.21 locus (Fig. 3f) and the distinct repression pattern of *CHCHD2* (Fig. 3g) indicate that the isogenic set of hESCs represents culture-adapted characteristics, at least at the transcriptome level (Fig. 3h).

### Alterations in chromatin accessibility in culture-adapted variants

The survival traits and *BCL2L1* expression evident in variants were not replicated in early-passaged iPSCs carrying the 20q11.21 gain^21^. Transcriptome profiles of iPSCs obtained from the Korea National Institute of Health (KNIH) (eight normal, four with 20q11.21 gain, including four iPSCs previously reported^21^ (Supplementary Fig. 3a) reinforced this consistency. Notably, the 20q11.21 gain in these iPSCs did not induce the expression of genes, such as *HM13, ID1, BCL2L1*, and *TPX2*, at the *20q11.21* locus (Supplementary Fig. 3b). In addition, two early-passaged hESC lines (KR1 and KR2: WA09 hESCs) from two independent laboratories at the Korea Research Institute Bioscience and Biotechnology (KRIBB) were examined. Intriguingly, the KR2 line with a 20q11.21 gain (Supplementary Fig. 3c) did not exhibit *BCL2L1* induction (Supplementary Fig. 3d) or resistance to YM155 or nocodazole (Noc) treatment (Supplementary Fig. 3e). Furthermore, the KR2 line maintained an intact TP53 status (Supplementary Fig. 3f).

**Fig. 4:**
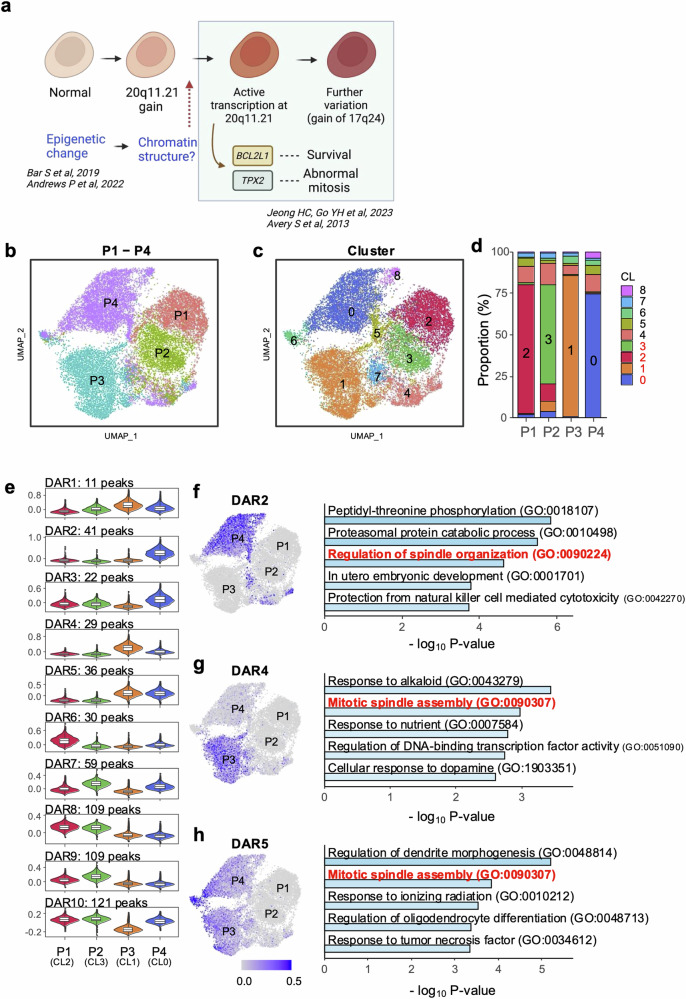
Alteration of chromatin accessibility in culture-adapted variants. **a** Scheme of chromatin structural alterations associated with culture-adapted variants. **b** Uniform manifold approximation and projection (UMAP) of scATAC-seq data from all the samples from P1 to P4. Each dot in the scatter plot represents single-cell chromatin accessibility. The four samples are color coded. **c** Clustering analysis based on chromatin accessibility. All the cells are grouped into 9 clusters and marked with numbers from 0 to 8. **d** The bar plot represents the proportion of a cluster for each sample. The major clusters are CL2 for P1, CL3 for P2, CL1 for P3, and CL0 for P4. These major clusters account for 80.6% of all cells. **e** Violin plots showing average open chromatin levels of differentially accessible regions (DARs) in major clusters. The y-axis represents the scaled average open chromatin levels of the DARs, which was calculated via the AddModuleScore() function. All DARs were clustered via k-means clustering with k = 10. The number of peaks belonging to a specific DAR are indicated. **f**–**h** Representative DARs corresponding to passages and Gene Ontology analysis of their target genes. UMAP shows the average level of open chromatin accessibility of the indicated DARs. The bar plot represents the top 5 Gene Ontology (GO) terms associated with putative target genes of the indicated DARs. The GO terms related to spindle assembly are marked in red. The p values of the enrichment were calculated via Metascape.

According to the scRNA data indicating active gene expression at 20q11.21 in later passages (Fig. 3e, f), an additional molecular event, such as epigenetic alterations^22,48^, could transcriptionally activate genes at this locus during in vitro culture. This event may directly induce the abnormal phenotypes of ‘culture-adapted variants’ (i.e., survival traits or abnormal mitosis) (Fig. 4a). Therefore, single-cell ATAC-seq was performed to monitor chromatin accessibility at the single-cell level. Major populations depending on these passages were distinctly clustered with varying chromatic accessibility. However, some minor populations existed in the overall distribution (Fig. 4b). Further classification revealed nine subclusters (Fig. 4c), and hESCs with different passage numbers formed their own major cluster (CL): CL2 in P1, CL3 in P2, CL1 in P3, and CL0 in P4.

**Fig. 5:**
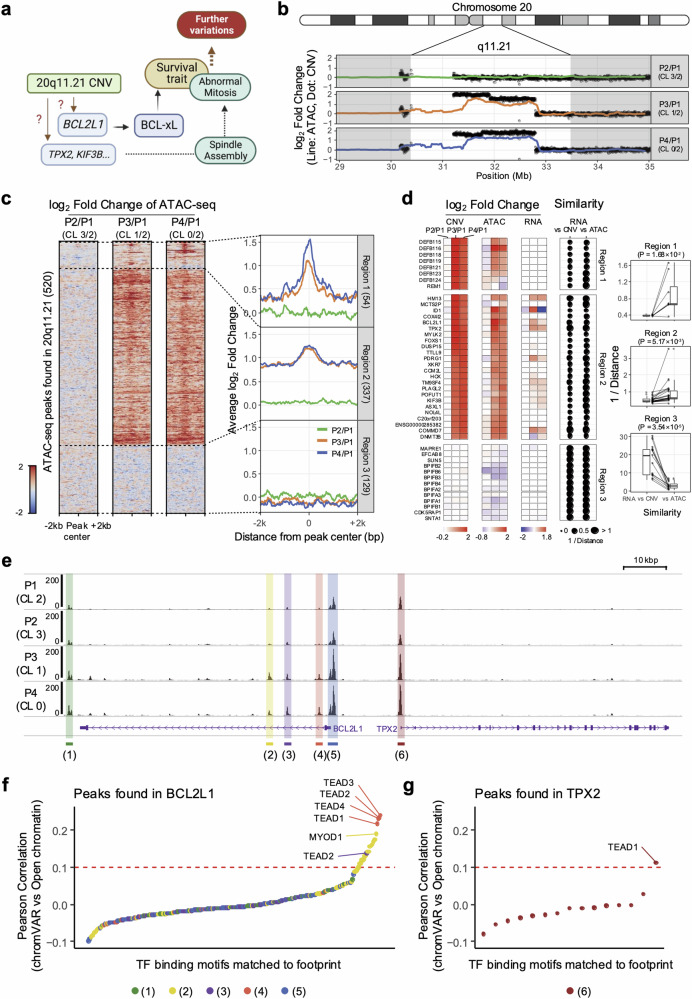
Epigenetic alteration affects genetic alterations in terms of gene expression. **a** Scheme of phenotypic variations driven by genetic and epigenetic alterations in culture-adapted cells. **b** ATAC-seq signal along with CNV around the 20q11.21 locus. The dots and lines represent the log_2_-fold changes against P1 in terms of the copy number and ATAC-seq read count, respectively. **c** Heatmaps representing the pattern of the log_2_-fold change in the ATAC-seq signal around all the peaks found at 20q11.21. The fold changes were calculated against the P1 major cluster (CL2). The line plots show the average log_2_-fold change around peaks found at the same genomic loci. Each region (Regions 1, 2, and 3) was separated by dotted lines. **d** Comparative analysis of similarities between CNV and RNA and between ATAC and RNA. The heatmaps indicate log_2_-fold changes in CNV, ATAC and RNA for each gene located at 20q11.21. The dot size represents the similarity score calculated by the inverse of the distance. The significance of the similarity score difference was calculated via paired *t* tests. **e** A representative scATAC-seq profile at the BCL2L1 and TPX2 loci. The *y*-axis value represents the normalized ATAC-seq read count. The DAR peaks are marked by color boxes and numbered. The location and direction of genes are shown at the bottom. **f**, **g** Pearson correlation of the transcription factor (TF) activity score (chromVAR) of the transcription factor and open chromatin level at the peak. TF binding motifs are ordered by their correlation values. The TEAD family of TF binding motifs was most highly correlated with the BCL2L1 locus [Fig. 5e (1) ~ (5)]. The TF with the highest correlation at the TPX2 locus [Fig. 5e (6)] was identified as TEAD1.

Overall, 567 differentially accessible regions (DARs) were identified in these major clusters. K-means clustering (k = 10) grouped all DARs into ten distinct clusters (Fig. 4d). For example, DAR2 and DAR4 chromatin accessibility was predominantly increased in major clusters of culture-adapted variants (i.e., P3 and P4). Thus, potential target genes were selected via associated cis-coaccessibility network (CCAN) analysis to investigate genes regulated by each DAR and their associations with specific phenotypes or cellular processes (Supplementary Fig. 4a). Similar to a previous report on methylation at the *CHCHD2* promoters in the variants^41^, the lack of an scATAC-seq profile at the *CHCHD2* promoter validated this analysis (Supplementary Fig. 4b). In this context, DARs were categorized into a total of 10 groups (Fig. 4e). DAR2 (Fig. 4f), 4, (Fig. 4g) and 5 (Fig. 4h) were generally associated with spindle assembly, whereas DAR6 (Supplementary Fig. 4c), 8 (Supplementary Fig. 4d), and 9 (Supplementary Fig. 4e) were frequently localized in normal hESCs (i.e., P1 and P2) without common gene ontology terms. In sharp contrast, DAR2 (primarily in P4), DAR4 (P3), and DAR5 (P3 and P4) were strongly associated with ‘spindle organization’.

These results suggest that an epigenetic change that alters chromatin structure-regulating genes associated with ‘spindle organization’ is induced in culture-adapted variants (i.e., P3 and P4). Consistently, aberrant mitosis with a lagging chromosome or chromosome bridge that results from abnormal spindle dynamics occurs in these culture-adapted variants^18^.

### Epigenetic alteration affects genetic alterations in terms of gene expression

We hypothesized that a molecular event to activate the chromatin structure of the 20q11.21 locus would transpire in hPSCs along with a 20q11.21 gain. This event would also promote the expression of crucial genes, such as *BCL2L1* (for survival) or *TPX2* (for spindle stabilization and abnormal mitosis), to achieve the typical cellular phenotypes of culture-adapted variants (i.e., survival traits and abnormal mitosis)^11,12,18^ (Fig. 5a). To verify this theory, subsequent studies were conducted to analyze the chromatin structure of loci that presented increased DNA copy numbers.

Open chromatin levels exhibit dynamic changes across the genome, but these changes are dependent on CNV changes in regions with significant variations (e.g., 20q11.21 in P3 and P4; 17q24.1 and 17q24.2 in P4; Fig. 5b, Supplementary Fig. 5b, c). This observation may be attributed to the nature of the ATAC-seq technique, which reads DNA derived from open chromatin regions and enables the measurement of chromatin accessibility. However, when the average open chromatin around the peaks at 20q11.21 was examined, three distinct patterns were observed (Fig. 5c): Pattern 1 (Region 1, chr20:30,400,000-31,502,846) presented a significant increase in open chromatin at the peak center; Pattern 2 (Region 2, chr20:31,502,846-32,804,612) presented an overall increase in open chromatin around ±2 kb of the peak center; and Pattern 3 (Region 3, chr20:32,804,612-33,500,000) presented no changes in CNV and open chromatin.

The broad increase in open chromatin observed approximately ±2 kb from the peak center in Region 2 is attributable to DNA copy number amplification. In contrast, the localized increase in open chromatin at the peak center suggests additional epigenetic alterations. Notably, genes located in Region 2 presented increased open chromatin spanning their gene bodies, with a further increase in open chromatin downstream of 1 kb from the transcription start site (TSS) (Supplementary Fig. 5a). This finding implies that gene copy amplification, including regulatory sequences, and additional downstream epigenetic alterations were apparent at the promoter region. Therefore, changes in CNV patterns, open chromatin, and gene expression were compared across protein-coding genes within the 20q11.21 locus to investigate the importance of downstream genetic and epigenetic alterations in gene expression (Fig. 5d).

Although there were individual gene-specific differences, patterns of changes in RNA expression in Regions 1 and 2 closely resembled the pattern of open chromatin changes rather than CNV changes. This observation suggests that gene expression alterations at the 20q11.21 locus are likely due to upstream epigenetic alterations. On the other hand, with respect to the 17q24 locus, chromatin structure alterations did not resemble RNA expression variations compared with copy number changes. (Supplementary Fig. 5d, e). This scenario implies that DNA copy number alterations drive chromatin structure changes at the 17q24 locus.

Next, Tn5 footprinting was performed within each cluster, and the TF binding motifs in the footprints were examined to identify the transcription factors (TFs) involved in *BCL2L1* and *TPX2* expression. While the loss of the CHCHD2-specific peak was evident in P3 and P4 hESCs (Supplementary Fig. 5f), the peaks were markedly elevated in the *BCL2L1* and *TPX2* regions (Fig. 5e). The Pearson correlation coefficient (PCC) between the number of open chromatin molecules at each peak location and the TF activity score in each cell was calculated at the single-cell level. The higher the correlation coefficient was, the greater the level of involvement of open chromatin at the peak location in the TF activity calculation, suggesting a greater probability that the TF is actively binding at the peak. Among various TF candidates, the TEAD family exhibited a strong correlation between open chromatin and TF activity at the promoters of *BCL2L1* (Fig. 5f) and *TPX2* (Fig. 5g). Moreover, the indicative power of the TEAD family activity score increased at P3 and P4 (Supplementary Fig. 5g). Consistently, a distinct increase in TEAD4 protein levels was observed in P3 and P4, alongside BCL-xL protein induction (Supplementary Fig. 5h). These findings suggest that the TEAD family of TFs, consistent with YAP activation (or the loss of Hippo signaling) in culture-adapted variants^17,35,40^, may be pivotal in achieving ‘culture-adapted phenotypes’.

## Discussion

Compared with adult stem cells or organoids, the unique cellular features of hPSCs, characterized by high sensitivity to DNA damage-induced apoptosis and robust DNA damage repair systems, contribute to well-maintained genome integrity^49^ and lower mutation rates during in vitro culture^7^. Long-term exposure to high oxygen concentrations during culture has been recently identified as a primary cause of spontaneous mutation^50^. Dominant-negative mutations in TP53, in which intact activation drives rapid apoptosis, are recurrent in hPSCs^15,16^. In contrast, recurrent CNVs at specific loci, especially 20q11.21 gain^10^, are relatively common even in early-passage iPSCs^21,51^. A recent meta-analysis of 107 different studies revealed that chromosomes 20, 12, 17, X, and 1 are prevalent hotspots for genetic abnormalities^52^. Despite emerging reports on altered cellular phenotypes, such as acquired survival traits^11–13^, aberrant mitosis^18^, aneuploidy^5,53^, and tumor development^54,55^, the underlying molecular processes for acquiring ‘culture-adapted phenotypes’ or minimum risk assessments remain poorly understood.

As previously described^7,50^, somatic hESC mutations were less evident in P2 hESCs (cultured over 100 passages), which presented normal copy numbers and wild-type TP53. However, somatic mutations, including COSMIC Tier 1 mutations, were drastically more abundant concurrently with TP53 mutations and 20q11.21 CNVs in P3 and P4 hESCs (Fig. 1). The amplification of somatic mutations and gains, especially at chromosome 1 after TP53 knockout (Fig. 2 and Supplementary Fig. 2g), implies that TP53 mutants undergo further genetic abnormalities. These results highlight the rationale for avoiding the use of rare TP53 mutant hPSCs in clinical applications.

On the other hand, hPSCs with 20q11.21 gain survival ability through *BCL2L1* expression. However, *BCL2L1* induction did not occur in iPSCs or hESCs with definitive 20q11.21 gain during the early passage^21^ (KR2 in Supplementary Fig. 3). Thus, gene expression at 20q11.21, which has biological consequences (i.e., survival traits or aberrant mitosis), requires additional molecular events. Multiomics analyses (i.e., scRNA-seq; Fig. 3 and scATAC-seq; Fig. 4) revealed that additional epigenetic events to open the 20q11.21 chromatin structure are necessary for the transcriptional activation of prominent genes at this locus (e.g., *BCL2L1* and *TPX2*) through TEAD upon promoter binding.

Interestingly, the absence of these genetic abnormalities and the similarity of single-cell transcriptome profiles to those of P1 hESCs in P2 hESCs (despite more than 100 passages) suggest that critical events during culture-adapted phenotype development potentially surpass the influence of passage number. This finding implies that TP53 mutations or 20q11.21 gain are likely more hazardous than the passage number alone. Thus, P3 and P4 hESCs are promising cellular models for monitoring biological consequences after these adverse events, regardless of unfeasible passage numbers. Notably, complete TP53 KO increased the number of somatic mutations and induced chromosomal gains, especially on chromosome 1, albeit without concurrent gains at 20q11.21 or 17q.24 (Fig. 2 and Supplementary Fig. 2). Finally, the current study raises questions about the reciprocal nature of these molecular abnormalities, particularly in hESCs (such as KR2) with a 20q11.21 gain that maintained the TP53 wild-type status (Supplementary Fig. 3f). The set of hESC pairs (i.e., KR1 and KR2) presents an intriguing model for monitoring the acquisition of ‘culture-adapted phenotypes’.

Given the significant safety concerns associated with genetic aberrations in hPSCs during in vitro culture, even at early passages, it is crucial not only to minimize the incidence of these variants but also to detect and eliminate them. While a few studies have attempted to address this issue by identifying biomarkers for transformed hPSCs^36,56^ or by inducing selective cell death^5,13^, challenges remain. The reproducibility of surface markers^57^ and the limited efficiency of selective cell death in genetic variants^5,13^ are still problematic. Therefore, further studies focusing on the characterization, early detection, and elimination of genetic variants are essential to ensuring the safety of hPSC-based cell therapies.

In conclusion, our findings emphasize that molecular mechanisms for epigenetic remodeling are crucial for transcriptionally activating genes at the 20q11.21 locus, which induces ‘culture-adapted phenotypes’ in hPSCs. On the basis of these results, TP53 mutation and 20q11.21 gain rather than the passage number should be regarded as hazardous events in hPSCs.

## Supplementary information

Supplementary Information

## Data Availability

All unique/stable reagents generated in this study will be freely available from the lead contact to academic researchers upon request. Whole-genome sequencing (WGS), RNA-Seq, scRNA-Seq data for P1, P2, P3, and P4 hESCs generated in this study have been deposited in the NCBI Sequence Read Archive (SRA) under the BioProject accession number PRJNA1020454 (link: https://dataview.ncbi.nlm.nih.gov/object/PRJNA1020454?reviewer=8qorbhrbs04mrmrmonmm5lek5d). ScATAC-Seq data for those four samples generated in this study have also been deposited in the NCBI SRA under the BioProject accession number PRJNA1020991 (link: https://dataview.ncbi.nlm.nih.gov/object/PRJNA1020991?reviewer=7m7slq0d272jmap4pjsvd2spp9).

## References

[1] Deinsberger, J., Reisinger, D. & Weber, B. Global trends in clinical trials involving pluripotent stem cells: a systematic multi-database analysis. *npj Regen. Med.* **5**, 1–13 (2020). 32983575 10.1038/s41536-020-00100-4 PMC7486930

[2] Jeong, H. C., Cho, S. J., Lee, M. O. & Cha, H. J. Technical approaches to induce selective cell death of pluripotent stem cells. *Cell Mol. Life Sci*. 10.1007/s00018-017-2486-0 (2017). 10.1007/s00018-017-2486-0 PMC11107638 28246701

[3] Mandai, M. et al. Autologous induced stem-cell-derived retinal cells for macular degeneration. *N. Engl. J. Med.* **376**, 1038–1046 (2017). 28296613 10.1056/NEJMoa1608368

[4] Andrews, P. W. et al. Assessing the safety of human pluripotent stem cells and their derivatives for clinical applications. *Stem Cell Rep.* **9**, 1–4 (2017). 10.1016/j.stemcr.2017.05.029 PMC7206225 28700896

[5] Ben-David, U. et al. Aneuploidy induces profound changes in gene expression, proliferation and tumorigenicity of human pluripotent stem cells. *Nat. Commun.* **5**, 4825 (2014). 25198699 10.1038/ncomms5825

[6] Lund, R. J., Narva, E. & Lahesmaa, R. Genetic and epigenetic stability of human pluripotent stem cells. *Nat. Rev. Genet* **13**, 732–744 (2012). 22965355 10.1038/nrg3271

[7] Kuijk, E. et al. The mutational impact of culturing human pluripotent and adult stem cells. *Nat. Commun.* **11**, 2493 (2020). 32427826 10.1038/s41467-020-16323-4 PMC7237696

[8] Liu, J. C. et al. High mitochondrial priming sensitizes hESCs to DNA-damage-induced apoptosis. *Cell Stem Cell* **13**, 483–491 (2013). 23954752 10.1016/j.stem.2013.07.018 PMC4109647

[9] Dumitru, R. et al. Human embryonic stem cells have constitutively active Bax at the Golgi and are primed to undergo rapid apoptosis. *Mol. Cell* **46**, 573–583 (2012). 22560721 10.1016/j.molcel.2012.04.002 PMC3372694

[10] International Stem Cell, I. et al. Screening ethnically diverse human embryonic stem cells identifies a chromosome 20 minimal amplicon conferring growth advantage. *Nat. Biotechnol.* **29**, 1132–1144, (2011). 22119741 10.1038/nbt.2051 PMC3454460

[11] Nguyen, H. T. et al. Gain of 20q11.21 in human embryonic stem cells improves cell survival by increased expression of Bcl-xL. *Mol. Hum. Reprod.* **20**, 168–177 (2014). 24217388 10.1093/molehr/gat077

[12] Avery, S. et al. BCL-XL mediates the strong selective advantage of a 20q11.21 amplification commonly found in human embryonic stem cell cultures. *Stem Cell Rep.* **1**, 379–386 (2013). 10.1016/j.stemcr.2013.10.005 PMC3841249 24286026

[13] Cho, S. J. et al. Selective elimination of culture-adapted human embryonic stem cells with BH3 mimetics. *Stem Cell Rep.* **11**, 1244–1256 (2018). 10.1016/j.stemcr.2018.09.002 PMC6235677 30293852

[14] Amir, H. et al. Spontaneous single-copy loss of TP53 in human embryonic stem cells markedly increases cell proliferation and survival. *Stem Cells* **35**, 872–885 (2017). 27888558 10.1002/stem.2550

[15] Merkle, F. T. et al. Human pluripotent stem cells recurrently acquire and expand dominant negative P53 mutations. *Nature* **545**, 229–233 (2017). 28445466 10.1038/nature22312 PMC5427175

[16] Lezmi, E., Jung, J. & Benvenisty, N. High prevalence of acquired cancer-related mutations in 146 human pluripotent stem cell lines and their differentiated derivatives. *Nat. Biotechnol.*10.1038/s41587-023-02090-2 (2024). 10.1038/s41587-023-02090-2 38195986

[17] Kim, Y. J. et al. TPX2 prompts mitotic survival via the induction of BCL2L1 through YAP1 protein stabilization in human embryonic stem cells. *Exp. Mol. Med*. 10.1038/s12276-022-00907-9 (2023). 10.1038/s12276-022-00907-9 PMC9898288 36596852

[18] Jeong, H. C. et al. TPX2 Amplification-Driven Aberrant Mitosis in Culture Adapted Human Embryonic Stem Cells with gain of 20q11.21. *Stem Cell Rev. Rep.*10.1007/s12015-023-10514-4 (2023). 10.1007/s12015-023-10514-4 36862329

[19] Zhang, J. et al. Anti-apoptotic mutations desensitize human pluripotent stem cells to mitotic stress and enable aneuploid cell survival. *Stem Cell Rep.* **12**, 557–571 (2019). 10.1016/j.stemcr.2019.01.013 PMC6411485 30773485

[20] Halliwell, J., Barbaric, I. & Andrews, P. W. Acquired genetic changes in human pluripotent stem cells: origins and consequences. *Nat. Rev. Mol. Cell Biol.* **21**, 715–728 (2020). 32968234 10.1038/s41580-020-00292-z

[21] Jo, H. Y. et al. Functional in vivo and in vitro effects of 20q11.21 genetic aberrations on hPSC differentiation. *Sci. Rep.* **10**, 18582 (2020). 33122739 10.1038/s41598-020-75657-7 PMC7596514

[22] Bar, S. & Benvenisty, N. Epigenetic aberrations in human pluripotent stem cells. *EMBO J*. **38**, 10.15252/embj.2018101033 (2019). 10.15252/embj.2018101033 PMC6576196 31088843

[23] Konki, M. et al. Epigenetic silencing of the key antioxidant enzyme catalase in karyotypically abnormal human pluripotent stem cells. *Sci. Rep.* **6**, 22190 (2016). 26911679 10.1038/srep22190 PMC4766493

[24] Weissbein, U., Plotnik, O., Vershkov, D. & Benvenisty, N. Culture-induced recurrent epigenetic aberrations in human pluripotent stem cells. *PLoS Genet* **13**, e1006979 (2017). 28837588 10.1371/journal.pgen.1006979 PMC5587343

[25] Gao, J. J. et al. 3D clusters of somatic mutations in cancer reveal numerous rare mutations as functional targets. *Genome Med* **9**, 4 (2017). 10.1186/s13073-016-0393-x. 10.1186/s13073-016-0393-x PMC5260099 28115009

[26] Stuart, T., Srivastava, A., Madad, S., Lareau, C. A. & Satija, R. Single-cell chromatin state analysis with Signac. *Nat. Methods* **18**, 1333–1341 (2021). 34725479 10.1038/s41592-021-01282-5 PMC9255697

[27] Pliner, H. A. et al. Cicero predicts cis-regulatory DNA interactions from single-cell chromatin accessibility data. *Mol. Cell* **71**, 858–871.e858 (2018). 30078726 10.1016/j.molcel.2018.06.044 PMC6582963

[28] Zhou, Y. et al. Metascape provides a biologist-oriented resource for the analysis of systems-level datasets. *Nat. Commun.* **10**, 1523 (2019). 30944313 10.1038/s41467-019-09234-6 PMC6447622

[29] Schep, A. N., Wu, B., Buenrostro, J. D. & Greenleaf, W. J. chromVAR: inferring transcription-factor-associated accessibility from single-cell epigenomic data. *Nat. Methods* **14**, 975–978 (2017). 28825706 10.1038/nmeth.4401 PMC5623146

[30] Fornes, O. et al. JASPAR 2020: update of the open-access database of transcription factor binding profiles. *Nucleic Acids Res.* **48**, D87–D92 (2020). 31701148 10.1093/nar/gkz1001 PMC7145627

[31] Zhang, Y. et al. Model-based analysis of ChIP-Seq (MACS). *Genome Biol.* **9**, R137 (2008). 18798982 10.1186/gb-2008-9-9-r137 PMC2592715

[32] Li, Z. et al. Identification of transcription factor binding sites using ATAC-seq. *Genome Biol.* **20**, 45 (2019). 30808370 10.1186/s13059-019-1642-2 PMC6391789

[33] Ramirez, F. et al. deepTools2: a next generation web server for deep-sequencing data analysis. *Nucleic Acids Res.* **44**, W160–165, (2016). 27079975 10.1093/nar/gkw257 PMC4987876

[34] Spits, C. et al. Recurrent chromosomal abnormalities in human embryonic stem cells. *Nat. Biotechnol.* **26**, 1361–1363 (2008). 19029912 10.1038/nbt.1510

[35] Price, C. J. et al. Genetically variant human pluripotent stem cells selectively eliminate wild-type counterparts through YAP-mediated cell competition. *Dev. Cell* **56**, 2455–2470 e2410 (2021). 34407428 10.1016/j.devcel.2021.07.019 PMC8443275

[36] Markouli, C. et al. Gain of 20q11.21 in human pluripotent stem cells impairs TGF-beta-dependent neuroectodermal commitment. *Stem Cell Rep.* **13**, 163–176 (2019). 10.1016/j.stemcr.2019.05.005 PMC6627003 31178415

[37] Ben-David, U. Genomic instability, driver genes and cell selection: projections from cancer to stem cells. *Biochim. Biophys. Acta* **1849**, 427–435 (2015). 25132386 10.1016/j.bbagrm.2014.08.005

[38] Narva, E. et al. High-resolution DNA analysis of human embryonic stem cell lines reveals culture-induced copy number changes and loss of heterozygosity. *Nat. Biotechnol.* **28**, 371–377 (2010). 20351689 10.1038/nbt.1615

[39] Martins-Taylor, K. et al. Recurrent copy number variations in human induced pluripotent stem cells. *Nat. Biotechnol.* **29**, 488–491 (2011). 21654665 10.1038/nbt.1890

[40] Weissbein, U. et al. Genome-wide screen for culture adaptation and tumorigenicity-related genes in human pluripotent stem cells. *iScience* **11**, 398–408 (2019). 30660107 10.1016/j.isci.2018.12.031 PMC6348297

[41] Kim, J. et al. Epigenetic repression of CHCHD2 enhances survival from single cell dissociation through attenuated Rho A kinase activity. *Cell Mol. Life Sci.* **81**, 38 (2024). 38214772 10.1007/s00018-023-05060-8 PMC10787008

[42] Tate, J. G. et al. COSMIC: the catalogue of somatic mutations in cancer. *Nucleic Acids Res.* **47**, D941–D947 (2019). 30371878 10.1093/nar/gky1015 PMC6323903

[43] Lee, M. O. et al. Inhibition of pluripotent stem cell-derived teratoma formation by small molecules. *Proc. Natl. Acad. Sci. USA* **110**, E3281–E3290 (2013). 23918355 10.1073/pnas.1303669110 PMC3761568

[44] Szklarczyk, D. et al. STRING v11: protein-protein association networks with increased coverage, supporting functional discovery in genome-wide experimental datasets. *Nucleic Acids Res.* **47**, D607–D613 (2019). 30476243 10.1093/nar/gky1131 PMC6323986

[45] Lane, D. P. Cancer. p53, guardian of the genome. *Nature* **358**, 15–16 (1992). 1614522 10.1038/358015a0

[46] Villa-Diaz, L. G., Ross, A. M., Lahann, J. & Krebsbach, P. H. Concise review: the evolution of human pluripotent stem cell culture: from feeder cells to synthetic coatings. *Stem Cells* **31**, 1–7 (2013). 23081828 10.1002/stem.1260 PMC3537180

[47] Krivec, N., Ghosh, M. S. & Spits, C. Gains of 20q11.21 in human pluripotent stem cells: Insights from cancer research. *Stem Cell Rep.* **19**, 11–27 (2024). 10.1016/j.stemcr.2023.11.013 PMC10828824 38157850

[48] Andrews, P. W. et al. The consequences of recurrent genetic and epigenetic variants in human pluripotent stem cells. *Cell Stem Cell* **29**, 1624–1636 (2022). 36459966 10.1016/j.stem.2022.11.006

[49] Weissbein, U., Benvenisty, N. & Ben-David, U. Quality control: genome maintenance in pluripotent stem cells. *J. Cell Biol.* **204**, 153–163 (2014). 24446481 10.1083/jcb.201310135 PMC3897183

[50] Thompson, O. et al. Low rates of mutation in clinical grade human pluripotent stem cells under different culture conditions. *Nat. Commun.* **11**, 1528 (2020). 32251294 10.1038/s41467-020-15271-3 PMC7089967

[51] Park, J. W., Bae, S. J., Yun, J. H., Kim, S. & Park, M. Assessment of genetic stability in human induced pluripotent stem cell-derived cardiomyocytes by using droplet digital PCR. *Int. J. Mol. Sci.* **25**, 10.3390/ijms25021101 (2024). 10.3390/ijms25021101 PMC10815998 38256178

[52] Assou, S. et al. Recurrent genetic abnormalities in human pluripotent stem cells: definition and routine detection in culture supernatant by targeted droplet digital PCR. *Stem Cell Rep.* **14**, 1–8 (2020). 10.1016/j.stemcr.2019.12.004 PMC6962701 31902703

[53] Na, J., Baker, D., Zhang, J., Andrews, P. W. & Barbaric, I. Aneuploidy in pluripotent stem cells and implications for cancerous transformation. *Protein Cell* **5**, 569–579 (2014). 24899134 10.1007/s13238-014-0073-9 PMC4130921

[54] Moon, S. H. et al. Effect of chromosome instability on the maintenance and differentiation of human embryonic stem cells in vitro and in vivo. *Stem Cell Res.* **6**, 50–59 (2011). 20920899 10.1016/j.scr.2010.08.006

[55] Yamamoto, T. et al. Correlation between genetic abnormalities in induced pluripotent stem cell-derivatives and abnormal tissue formation in tumorigenicity tests. *Stem Cells Transl. Med.* **11**, 527–538 (2022). 35445254 10.1093/stcltm/szac014 PMC9154342

[56] Herszfeld, D. et al. CD30 is a survival factor and a biomarker for transformed human pluripotent stem cells. *Nat. Biotechnol.* **24**, 351–357 (2006). 16501577 10.1038/nbt1197

[57] Harrison, N. J. et al. CD30 expression reveals that culture adaptation of human embryonic stem cells can occur through differing routes. *Stem Cells* **27**, 1057–1065 (2009). 19415777 10.1002/stem.41 PMC2860760

