# Supplementary material for: Longitudinal analysis of genetic and epigenetic changes in human pluripotent stem cells in the landscape of culture-induced abnormality
Source: Exp Mol Med. 2024 Nov 1;56(11):2409–22. doi: 10.1038/s12276-024-01334-8 (PMC11612254; doi:10.1038/s12276-024-01334-8)
Supplement: Supplementary file 1 — Supplementary Information [file 12276_2024_1334_MOESM1_ESM.pdf]

Supplementary Information

This PDF file includes :

Supplementary Figures

Supplementary Material and Methods

Supplementary Figure legends

Supplementary Material and Methods

Reagents and immunoblotting

The primary antibodies against β-actin (#sc-47778), Vinculin(sc-25336), and p53(sc-126). For immunoblotting, cell lysates were extracted with RIPA buffer supplemented with a 1% protease inhibitor cocktail and 0.1% sodium orthovanadate, incubated for 1-hour on ice and centrifuged for extraction. The concentration of total protein was quantified by the BCA protein assay kit (#23225, Thermo ScientificTM). Approximately 15μg of total protein were separated on 10% concentrations of SDS-PAGE, then transferred to the PVDF membrane. Membrane with protein was blocked with 5% skim milk in TBS-T (Tris-buffered saline with 0.1% Tween-20) for 1 hour and then washed by TBS-T for every 5 minutes three times. The membrane was incubated with the primary antibody in TBS-T (1:1000) with 0.1% sodium azide overnight, 4°C. The incubated membrane was washed for 5 minutes three times with TBS-T. The membrane was incubated with HRP-conjugated secondary antibody (Jackson Immunoresearch Laboratories) in TBS-T (1:10000) for 1 hour, room temperature. The incubated membrane was washed for 15 minutes three times with TBS-T. Immunoreactivity was detected by Chemi-Doc using WEST-QueenTM (#16026, iNtRON Biotechnology) kit. The band intensity was measured using Fusion FX software and normalized with the loading control.

Live cell imaging and Cell Death Assay

Cell population after Nutlin3a treatment was collected and analyzed by FACS Calibur (BD Bioscience). The cell image of bright field was captured by Light channel optical microscope (Olympus, Tokyo, Japan, CKX-41) or JuLI^{TM} Stage (NanoEntek, Seoul, Korea). For cell death assay, live cell imaging by JULI-stage with SYTOX green (ThermoFisher, S7020) 100nM staining. The fluorescence intensity of SYTOX green after Nocodazole or YM155 treatment

was collected and analyzed in accordance with the manufacturer's protocol using JuLI^{TM}STAT , then normalized by total cell area.

Supplementary Figure legends

Supplementary Fig. 1 (a) Identification of gain of 20q11.21 and 17q24.1/2 in P3 and/or P4 hESCs. Copy number variations (CNVs) were detected in P2, P3 and P4 hESCs in comparison to P1 hESC using CNVkit, with a cutoff of 2-fold change, based on whole-genome sequence data. The x-axis indicates log2 ratio of copy number. The y-axis indicates chromosomal position (Mb). **(b)** The number of somatic SNVs in genomic features by point mutation type. Left panel indicates SNVs in P3 compared to P1, and right panel indicates P4 SNVs. Point mutation types were classified to C>T, C>G, C>A, T>G, T>A, and T>C. **(c)** The number of somatic intergenic, intronic, and missense SNVs in P2, P3, and P4 hESCs by point mutation type. (**d**) The chromosomal distribution of somatic SNVs in P2, P3, and P4 hESCs. The chromosomal distribution of somatic SNVs is illustrated by PhenoGram (http://visualization.ritchielab.org**).**

Supplementary Fig. 2 (a) Identification of genes with non-coding and functional effect-related SNVs in *TP53*-directed network of P3 and P4 hESCs. Genes directly interacting with TP53 were searched by the STRING database and a network was then created using CytoScape. In the TP53-directed network, genes with those SNVs were searched. In the network, bright blue circles represent genes with non-coding SNVs, while yellow circles indicate SNVs with a potential functional effect. SNVs with a potential functional effect encompass missense, nonsense, and splice-site SNVs. **(b)** Target site of gRNA for TP53KO and actual alterations are presented. **(c)** Sequence of altered TP53 genes in DNA binding domain. After Cas9 and gRNA introduced, 1 base pair was inserted to genomic sequence and frame shift occurred. **(d)** The number of somatic SNVs in genomic features between P1 hESC and TP53 KO cell. **(e)** Identification of genes with non-coding SNVs in *TP53*-directed network of TP53 KO. **(f)** Identification of amplification (or gain) of chromosome 1 in TP53 KO. CNVs were detected in P2, P3, P4, and TP53 KO hESCs in comparison to P1 hESC using CNVkit, with a cutoff of 2-

fold change, based on whole-genome sequence data. (**g**) Validation of gain of chromosome 1 in P1, P1-D1, and TP53 KO via CytoScan HD array (ThermoFisher). (**h**) Identification of gain of 20q11.21 and 17q24.1/2 in TP53 KO. No CNV was detected in TP53 KO.

Supplementary Fig. 3 (a) The list of iPSCs with or without gain at 20q11.21. **(b)** Gene expression of the 20q11.21 region in each cell line was indicated in S3A [n=6, multiple paired t-test. P-values of every t-test were non-significant (NS)]. **(c)** CNV in 20q11.21 region of P1, KR1, and KR2. KR1 and KR2 are provided by two independent laboratories at the Korea Research Institute Bioscience and Biotechnology (KRIBB). **(d)** BCL2L1 mRNA level of WiCell, P1, P4, and KR1 cells. **(e)** Cell death by 20nM YM155 of P1 and KR2 were tested by SYTOX staining. The intensity of SYTOX was detected by live imaging as described and divided by total cell area. **(f)** The mutation status of P1, KR1, KR2, and P4 in TP53 R175H region was tested by PCR and Sanger sequencing.

Supplementary Fig. 4 (a) Scheme of the functional analysis of differentially accessible regions from major clusters. **(b)** The open chromatin level of the promoter of CHCHD2 is indicated in each cell projected on the UMAP plot using the feature plot function. The VlnPlot below the UMAP plot shows the abundance of open chromatin in each cluster. **(c, d and e)** Characteristic of each DAR and functional enrichment of their target genes. The UMAP shows the amount of average open chromatin level of indicated DAR. The bar plot represents the top 5 GO terms related to putative target genes of indicated DAR.

Supplementary Fig. 5. (a) The heatmaps represent the patterns of $\log_2$ fold change of ATAC-seq signal around all protein coding genes found at 20q11.21. The line plots denote average log2 fold change. Each region corresponds to the region at Figure 5c. **(b)** The overall colocalization of CNV and ATAC-seq peak signals **(c)** ATAC-seq signal along with copy number variation at 17q24.1 and 17q24.2. The dots and lines represent log2 fold change against

P1 of copy number and ATAC-seq read count for each. **(d)** The heatmaps represent patterns of $\log_2$ fold change of ATAC-seq signal around all peaks found at 17q24.1 and 17q24.2. The line plots show average $\log_2$ fold change around peaks found at the same genomic loci. **(e)** Similarity comparison between CNV and RNA, and between ATAC and RNA. The heatmaps shows $\log_2$ fold changes of CNV, ATAC and RNA for each gene located at 17q24.1 and 17q24.2. The dot size represents similarity score calculated as inverse of distance. The significance of similarity score difference was calculated by paired t-test. **(f)** A representative scATAC-seq profile at the promoter of *CHCHD2*. The y-axis value represents the normalized ATAC-seq read count. The DAR peak is marked by a light purple color box. **(g)** AUC score of transcription factor activity score calculated by chromVAR. **(h)** Immunoblotting of BCL-xL and TEAD4 in P1-P4 hESCs. β-actin as the loading control.

Supplementary Fig. 1

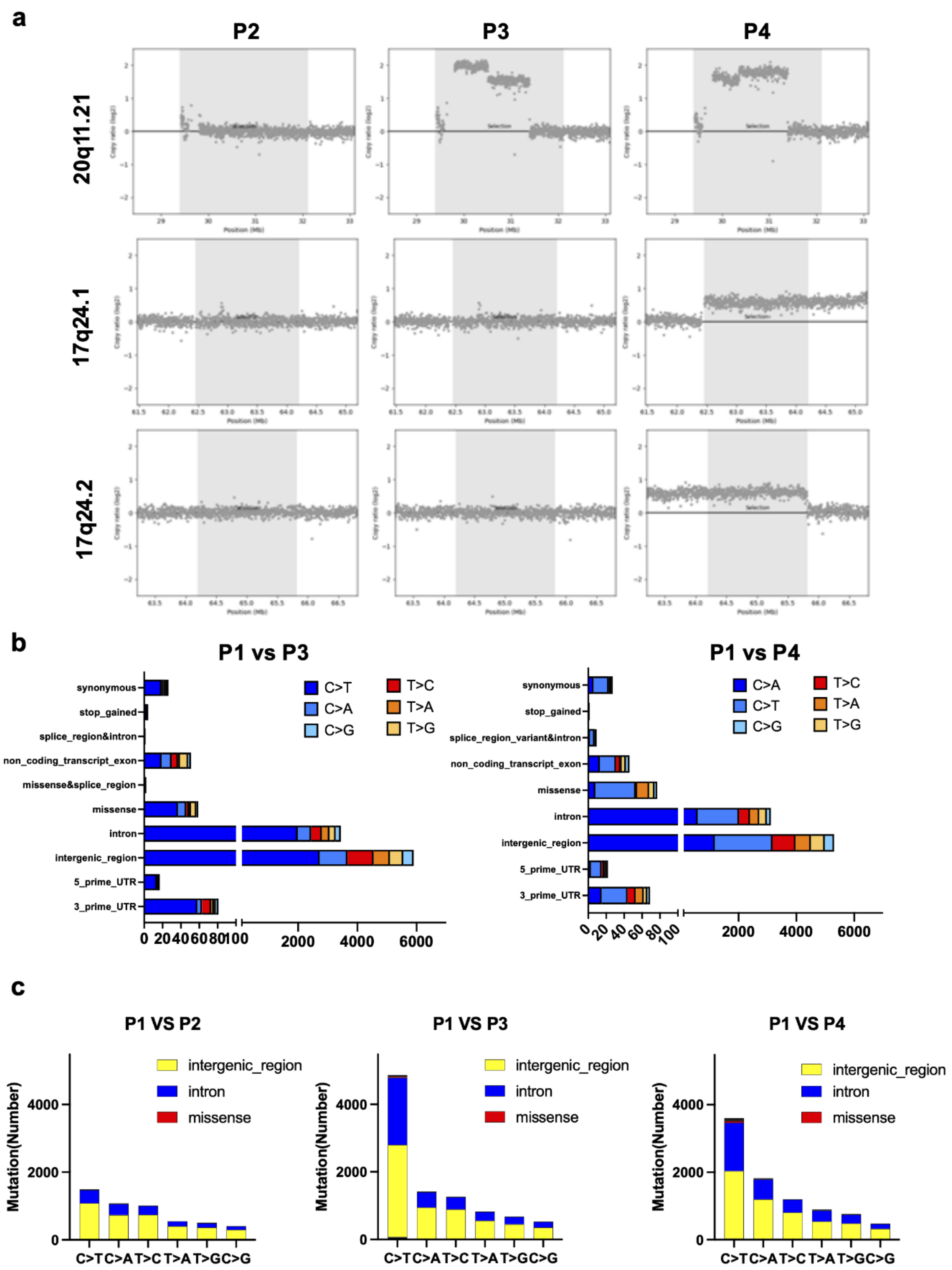

d

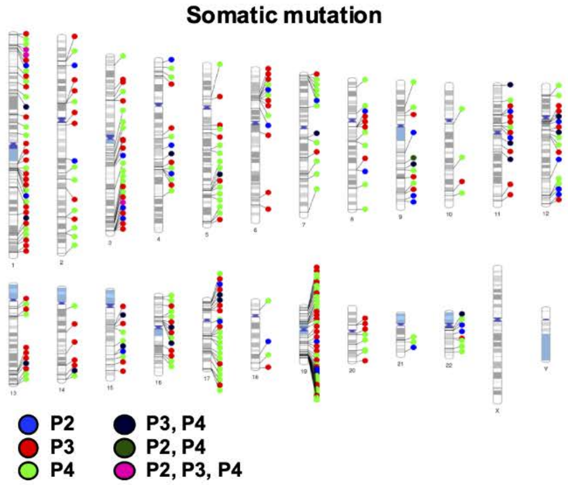
Somatic mutation
1
2
3
4
5
6
7
8
9
10
11
12
13
14
15
16
17
18
19
20
21
22
X
Y
P2
P3
P4
P3, P4
P2, P4
P2, P3, P4

Supplementary Fig. 2

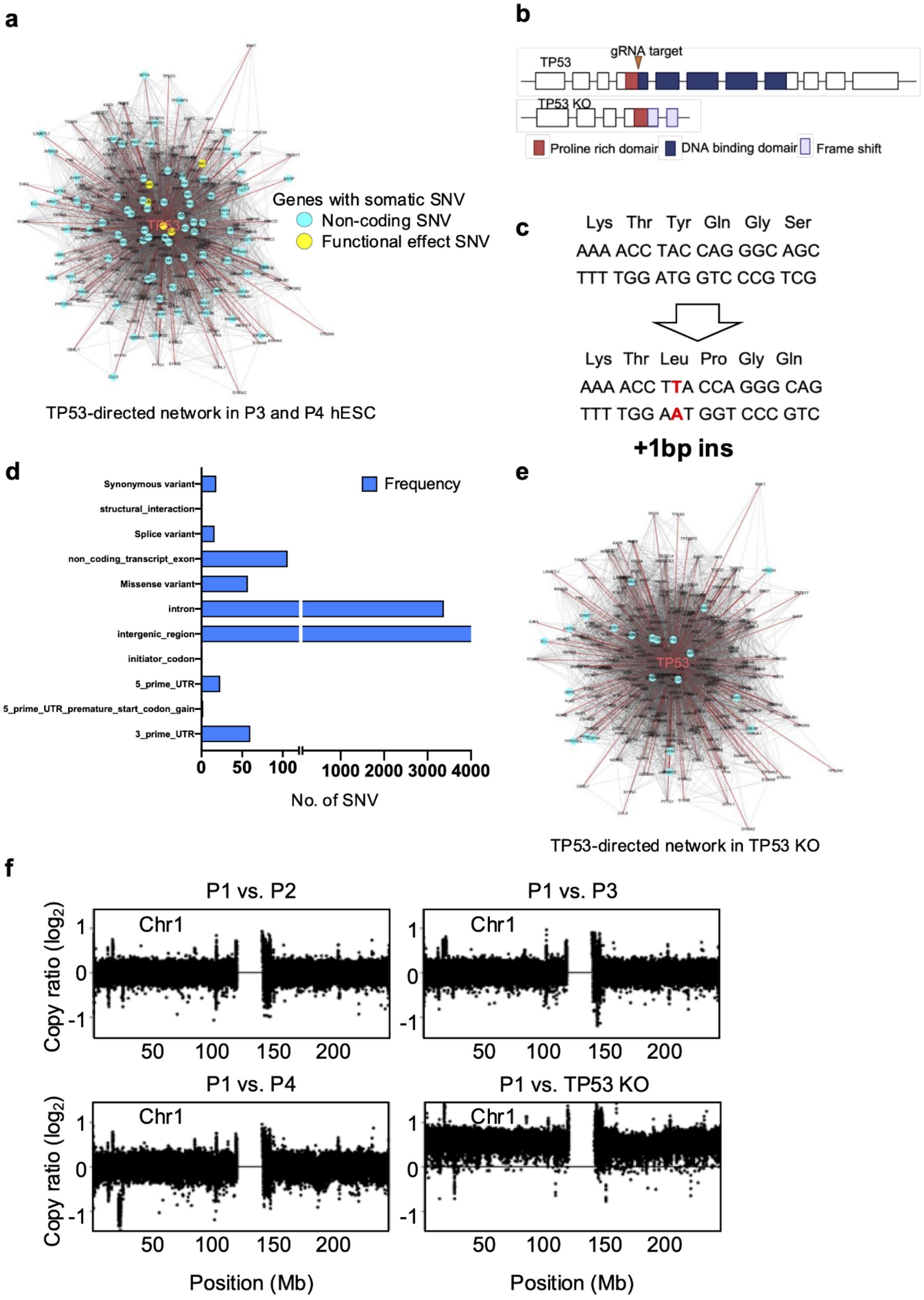

g

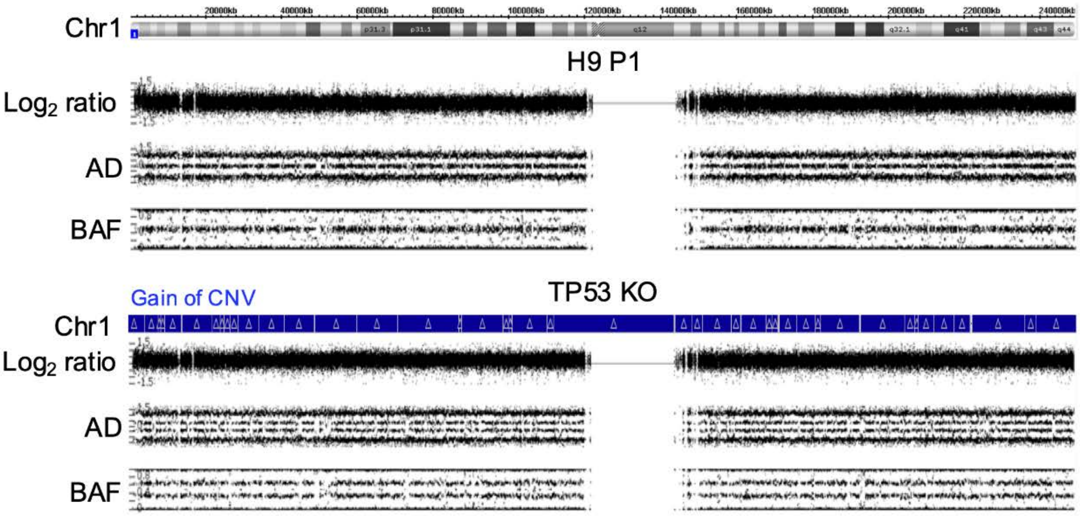
Chr1
H9 P1
Log2 ratio
AD
BAF
Gain of CNV
TP53 KO
Chr1
Log2 ratio
AD
BAF

h

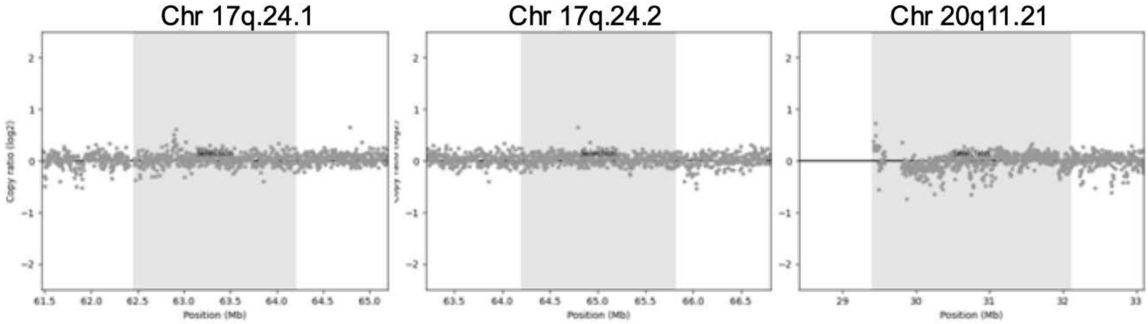
Chr 17q.24.1
Chr 17q.24.2
Chr 20q11.21
Copy ratio (log2)
Position (Mb)
Position (Mb)
Position (Mb)

Supplementary Fig. 3

a

Group	Cell Name	# passage	References (PMID)
Normal	KSCBi005-A	p20	33714852
	CMC-hiPSC-006	p13	-
	CMC-hiPSC-015	p15	-
	CMC-hiPSC-016	p15	-
	CMC-hiPSC-019	p18	-
	CMC-hiPSC-020	p12	-
20q11.21 CNV	KSCBi002-A	p34	33714852
	KSCBi002-A	p34	33714852
	KSCBi002-A-1	p26	33714852
	CMC-hiPSC-018	p15	-

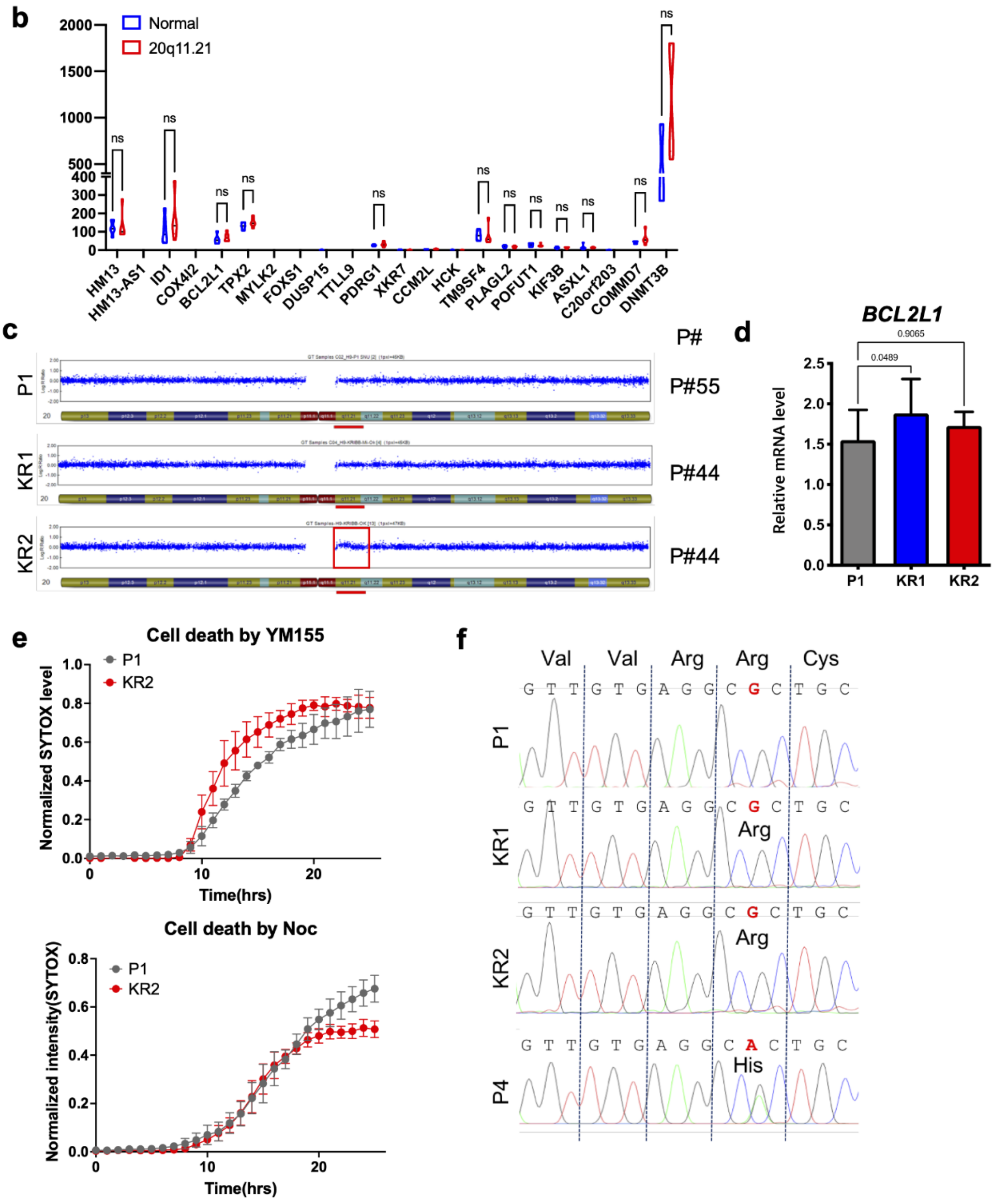

Supplementary Fig. 4

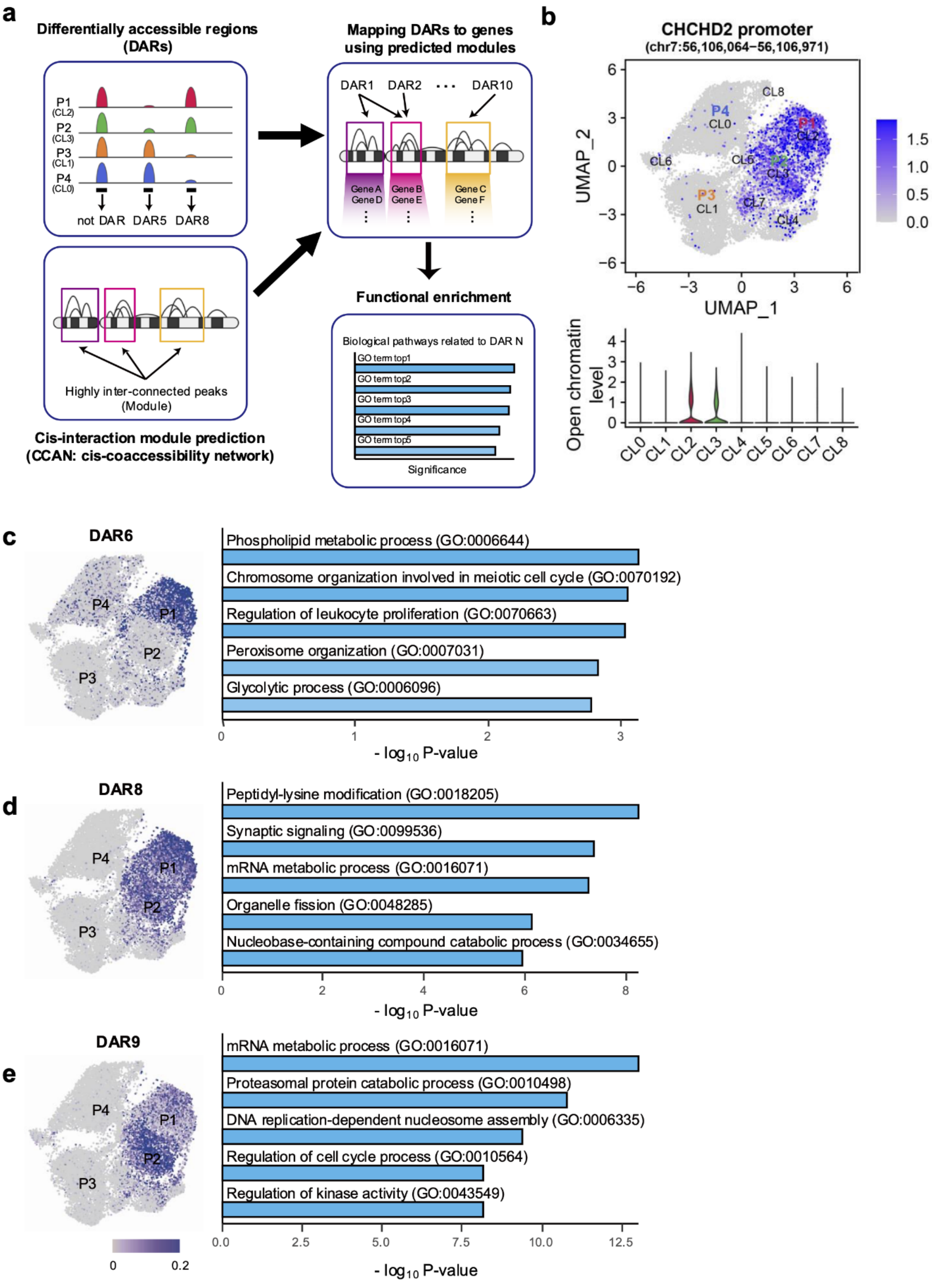

Supplementary Fig. 5

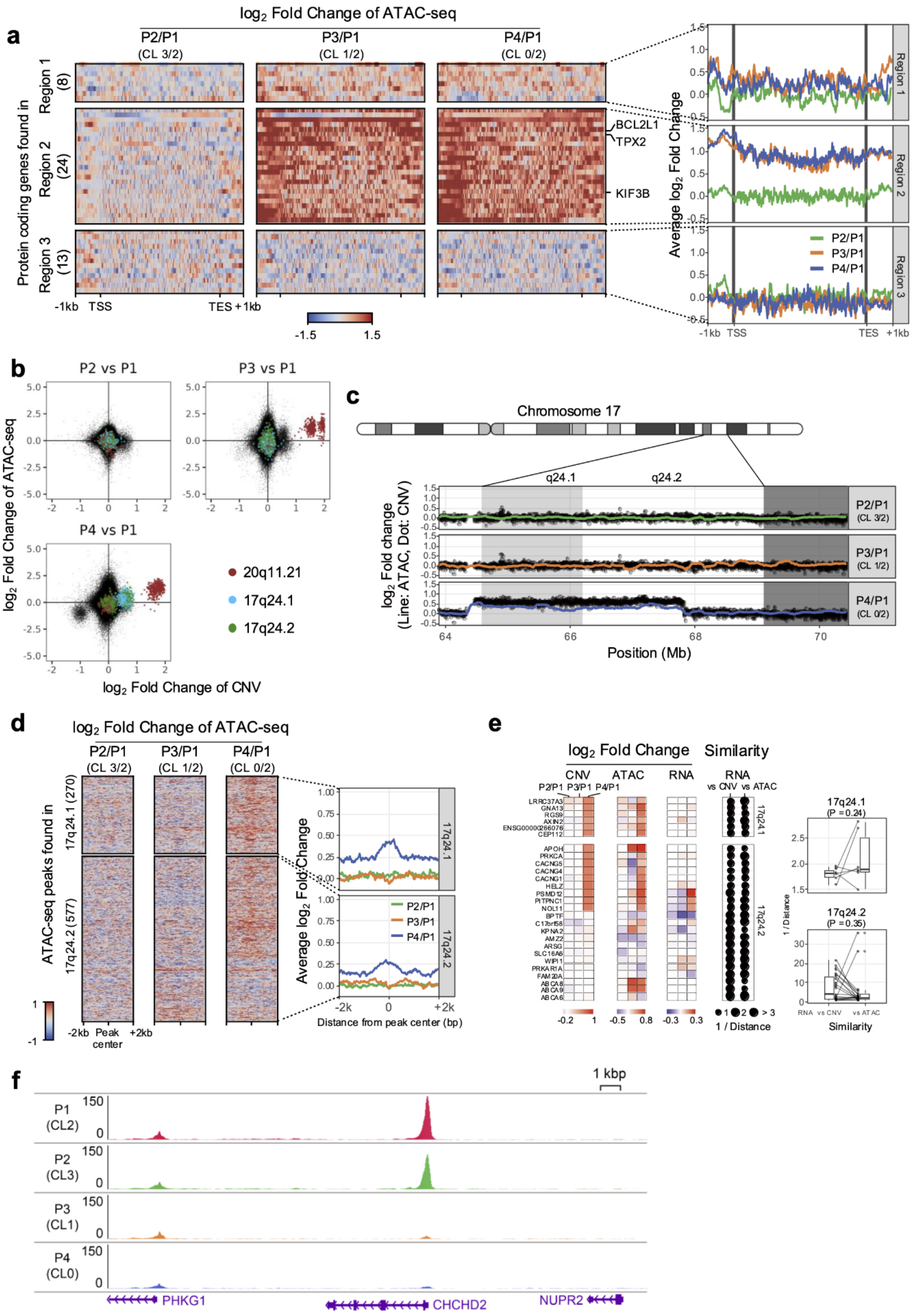

g

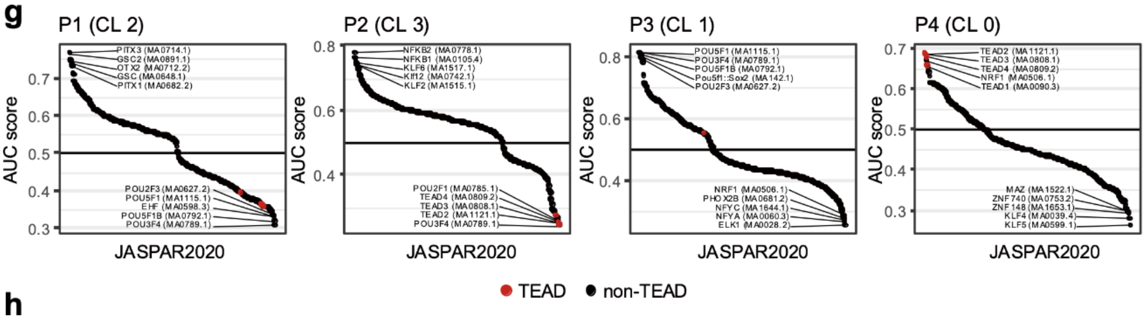
P1 (CL 2)
PITX3 (MA0714.1)
GSC2 (MA0891.1)
OTX2 (MA0712.2)
GSC (MA0648.1)
PITX1 (MA0682.2)
POU2F3 (MA0627.2)
POU5F1 (MA1115.1)
EHF (MA0598.3)
POU5F1B (MA0792.1)
POU3F4 (MA0789.1)
AUC score
0.7
0.6
0.5
0.4
0.3
JASPAR2020
P2 (CL 3)
NFKB2 (MA0778.1)
NFKB1 (MA0105.4)
KLF6 (MA1517.1)
Klf12 (MA0742.1)
KLF2 (MA1515.1)
POU2F1 (MA0785.1)
TEAD4 (MA0809.2)
TEAD3 (MA0808.1)
TEAD2 (MA1121.1)
POU3F4 (MA0789.1)
0.8
0.6
0.4
P3 (CL 1)
POU5F1 (MA1115.1)
POU3F4 (MA0789.1)
POU5F1B (MA0792.1)
Pou5f1::Sox2 (MA142.1)
POU2F3 (MA0627.2)
NRF1 (MA0506.1)
PHOX2B (MA0681.2)
NFYC (MA1644.1)
NFYA (MA0060.3)
ELK1 (MA0028.2)
P4 (CL 0)
TEAD2 (MA1121.1)
TEAD3 (MA0808.1)
TEAD4 (MA0809.2)
NRF1 (MA0506.1)
TEAD1 (MA0090.3)
MAZ (MA1522.1)
ZNF740 (MA0753.2)
ZNF148 (MA1653.1)
KLF4 (MA0039.4)
KLF5 (MA0599.1)
TEAD
non-TEAD

h

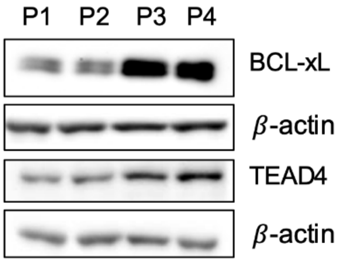
P1
P2
P3
P4
BCL-xL
β-actin
TEAD4
β-actin
